# Initial Complete Chloroplast Genomes of *Alchemilla* (Rosaceae): Comparative Analysis and Phylogenetic Relationships

**DOI:** 10.3389/fgene.2020.560368

**Published:** 2020-12-09

**Authors:** Peninah Cheptoo Rono, Xiang Dong, Jia-Xin Yang, Fredrick Munyao Mutie, Millicent A. Oulo, Itambo Malombe, Paul M. Kirika, Guang-Wan Hu, Qing-Feng Wang

**Affiliations:** ^1^CAS Key Laboratory of Plant Germplasm Enhancement and Specialty Agriculture, Wuhan Botanical Garden, Chinese Academy of Sciences, Wuhan, China; ^2^Sino-Africa Joint Research Center, Chinese Academy of Sciences, Wuhan, China; ^3^University of Chinese Academy of Sciences, Beijing, China; ^4^East African Herbarium, National Museums of Kenya, Nairobi, Kenya

**Keywords:** *Alchemilla argyrophylla*, *A. pedata*, chloroplast genome, phylogenetic, Rosaceae

## Abstract

The genus *Alchemilla* L., known for its medicinal and ornamental value, is widely distributed in the Holarctic regions with a few species found in Asia and Africa. Delimitation of species within *Alchemilla* is difficult due to hybridization, autonomous apomixes, and polyploidy, necessitating efficient molecular-based characterization. Herein, we report the initial complete chloroplast (cp) genomes of *Alchemilla*. The cp genomes of two African (*Afromilla*) species *Alchemilla pedata* and *Alchemilla argyrophylla* were sequenced, and phylogenetic and comparative analyses were conducted in the family Rosaceae. The cp genomes mapped a typical circular quadripartite structure of lengths 152,438 and 152,427 base pairs (bp) in *A. pedata* and *A. argyrophylla*, respectively. *Alchemilla* cp genomes were composed of a pair of inverted repeat regions (IRa/IRb) of length 25,923 and 25,915 bp, separating the small single copy (SSC) region of 17,980 and 17,981 bp and a large single copy (LSC) region of 82,612 and 82,616 bp in *A. pedata* and *A. argyrophylla*, respectively. The cp genomes encoded 114 unique genes including 88 protein-coding genes, 37 transfer RNA (tRNA) genes, and 4 ribosomal RNA (rRNA) genes. Additionally, 88 and 95 simple sequence repeats (SSRs) and 37 and 40 tandem repeats were identified in *A. pedata* and *A. argyrophylla*, respectively. Significantly, the loss of group II intron in *atpF* gene in *Alchemilla* species was detected. Phylogenetic analysis based on 26 whole cp genome sequences and 78 protein-coding gene sequences of 27 Rosaceae species revealed a monophyletic clustering of *Alchemilla* nested within subfamily Rosoideae. Based on a protein-coding region, negative selective pressure (Ka/Ks < 1) was detected with an average Ka/Ks value of 0.1322 in *A. argyrophylla* and 0.1418 in *A. pedata*. The availability of complete cp genome in the genus *Alchemilla* will contribute to species delineation and further phylogenetic and evolutionary studies in the family Rosaceae.

## Introduction

The genus *Alchemilla* L. (Rosaceae), composed of more than 1,000 species, is important for its ornamental and medicinal values (Kaya et al., 2012; Boroja et al., 2018). It is widely distributed in the Holarctic region with high species richness in west Eurasia and few species found in montane tropical Asia, Madagascar, South Africa, and East African mountains (Izmailow, 1981; Fröhner, 1995; Gehrke et al., 2008). *Alchemilla*, together with *Aphanes* and *Lachemilla*, is classified under subfamily Rosoideae, tribe Potentilleae and subtribe Alchemillinae (Rothmaler, 1937; Kalkman, 2004; Notov and Kusnetzova, 2004; Soják, 2008; Xiang et al., 2016). Initially, *Alchemilla* was categorized under tribe Sanguisorbinae by Hutchinson (1964) due to superficial similarity of floral traits but was later reclassified as Potentilleae following Schulze-Menz’s (1964) observational concern on the anther structure and further confirmation through molecular characterization using nuclear ribosomal DNA and *trnL/F* region of chloroplast (cp) DNA (Eriksson et al., 2003). Morphologically, *Alchemilla* is distinguished from other Rosaceae genera by the silvery-silky white hair covering on the stems and the leaf surface, achene fruits that are hidden within the calyx tube, inconspicuous individuality, and small but fairly showy inflorescence (Graham, 1960; Faghir et al., 2014; Gehrke et al., 2016). Notwithstanding, circumscription within the genus remain poorly understood due to hybridization, autonomous apomixes, and polyploidization dominant in *Alchemilla* species (Izmailow, 1982; Czapik, 1996; Gehrke et al., 2008). The genus *Alchemilla* is commonly used as perfect example of apomictic traits combined with morphological polymorphism in Rosaceae (Czapik, 1996; Bicknell and Koltunow, 2004; Hayirhoglu-Ayaz et al., 2006; Salamone et al., 2013). This has resulted in the existence of diverse micro-species and species complexes with variable indumentum, unstable flower characteristic structure, and heteroblastic plasticity, making *Alchemilla* a taxonomically difficult group (Hörandl, 2004; Hayirhoglu-Ayaz et al., 2006; Lundberg et al., 2009). This necessitated the need for complete cp genome in the genus *Alchemilla* to help resolve the taxonomic and phylogenetic uncertainties between its species.

Chloroplast (cp) is found in the cytoplasmic matrix of a plant cell and plays significant roles in photosynthesis, carbon fixation, and synthesis of starch, fatty acids, and amino acids (Daniell et al., 2016). It is a semi-autonomous organelle, similar to the nuclei and the mitochondria, essential in the transfer and expression of the plant’s genetic material (Wolfe et al., 1987). The cp has its own double-stranded circular genome whose size in most terrestrial plants ranges between 120 and 180 kb, encoding about 110–130 different genes in a highly conserved order (Shinozaki et al., 1986; Liu et al., 2017). Based on its genome content and the ultrastructure features, the cp traces its origin to free-living cyanobacteria through a single event of endosymbiosis (Gray, 1989; Keeling, 2004). The cp genome has a characteristic quadripartite structure comprising two identical copies of inverted repeat (IRa/IRb) regions separated by the large single copy (LCS) and small single copy (SSC) regions (Zhao M.-L. et al., 2018). The two IR regions are paramount in defining the size and structure of the cp genome of land plants (Palmer et al., 1988). Variations in genome size could consequently be due to expansion/contraction or loss of one of the IR regions in some species. For instance, the loss of one copy of IR in *Taxus chinensis* var. *mairea* resulted in the reduction of its genome size (Zhang et al., 2014). The uniqueness of the cp is evident in its maternal inheritance, small size, conserved sequences, and simple structure (Kress et al., 2005; Parks et al., 2009; Liu et al., 2017). The evolutionary process in angiosperms is dependent on the conserved structure, gene content, and organization of the cp genome (Doyle et al., 1992; Saski et al., 2005). This makes it an appropriate candidate for plant taxonomy, and comparative genomic and evolutionary studies. The advent of the next-generation DNA sequencing technology (Shendure and Ji, 2008) has magnified the rate of cp genome sequencing reports since the process is simpler, cost-effective and fast, resulting in the expansion of the cp genetics and genomics (Daniell et al., 2016). Since the report of the first cp genome sequence of tobacco (*Nicotiana tabacum*) (Shinozaki et al., 1986), several species in the Rosaceae have had their complete cp genome deposited to the National Center for Biotechnology Information (NCBI) organelle genome database^1^ (Salamone et al., 2013; Zhao Y. et al., 2018; Wang et al., 2020). However, none of the *Alchemilla* species genome has been sequenced to date.

In this study, the initial cp genomes of the genus *Alchemilla* are reported in *Alchemilla pedata* and *Alchemilla argyrophylla*. First, we obtained the complete cp genome of the two species and characterized the structure, gene content, and organization of each genome. Second, we establish the codon usage frequencies, simple sequence repeats (SSRs), regions of high sequence divergence, and nucleotide substitution rates. Finally, phylogenetic position was evaluated by comparative analysis based on 24 complete cp genomes and 78 protein-coding gene (PCG) sequences of Rosaceae species. Our results provide a reference for the resolution of *Alchemilla* species classification and facilitate elucidation of evolutionary and phylogenetic relationships in Rosaceae.

## Materials and Methods

### DNA Extraction and Chloroplast Genome Sequencing

Plant materials of two *Alchemilla* species of *Alchemilla pedata* (voucher number SAJIT-001337) and *Alchemilla argyrophylla* (SAJIT-002399) were collected from Mt. Kenya, Kenya. Young leaves were sampled and immediately preserved using silica gel in plastic bags (Chase and Hills, 1991). The voucher specimens were deposited at the East African Herbarium (EA) in the National Museums of Kenya and at the Herbarium of Wuhan Botanical Garden, CAS (HIB) (China). The total genomic DNA of the two species was extracted from 0.5 g of the silica dried leaves using modified cetyltrimethylammonium bromide (CTAB) protocol (Doyle, 1991). Results were then sequenced based on the Illumina paired end technology platform at the Novogen Company (Beijing, China).

### Genome Assembly and Genome Annotation

Genome assembly was performed using GetOrganelle v1.6.2d with default parameters (Jin et al., 2020). The GetOrganelle first filtered plastid-like reads, conducted the *de novo* assembly, purified the assembly, and finally generated the complete plastid genomes (Camacho et al., 2009; Bankevich et al., 2012; Langmead and Salzberg, 2012). *K*-mer gradients for a mean and maximum 150-bp reads were set to as “-k 21, 45, 65, 85,105” for both species. Bandage (Wick et al., 2015) was used to visualize the final assembly graphs to authenticate the automatically generated plastid genome. The best fit k-mer of 45 was selected for use in a subsequent analysis of the genomes. The quality of the newly assembled genomes was evaluated on read level basis by aligning the trimmed raw reads to the *de novo* assemblies using Geneious mapper, Geneious version 9.1.4 (Kearse et al., 2012) with medium- to low-sensitivity option and iteration up to five times (Köhler et al., 2020). Gene annotation was conducted using Plastid Genome Annotator (PGA) (Qu et al., 2019) with an annotated plastome *Amborella trichopoda* (GenBank accession no. GCA_000471905.1) as the initial reference genome. Further annotation confirmation was performed with published genomes of *Fragaria virginiana* (JN884817) and *Fragaria vesca* subsp. *vesca* (KC507760) in the Rosacea family. Geneious was used to manually correct and complement problematic annotations. The whole genome circular map was drawn using Organelle Genome DRAW software (Lohse et al., 2013).

### Comparative Genome Analysis and Sequence Divergence

The cp genome sequence of the *A. pedata* and *A. argyrophylla* was compared with that of eight other Rosaceae species. This included *Dasiphora fruticosa* (MF683841), *Fragaria iinumae* (KC507759), *Fragaria nipponica* (KY769125), *Fragaria pentaphylla* (KY434061), *Fragaria orientalis* (KY769126), *Fragaria chiloensis* (JN884816), *F. virginiana* (JN884817), *Fragaria mandshurica* (KC507760), *F. vesca* (KC507760), *Rosa multiflora* (NC_039989), *Rosa odorata* var. *gigantea* (KF753637), and *Hagenia abyssinica* (KX008604) retrieved from GenBank database. The mVISTA (Mayor et al., 2000) program under the shuffle-LAGAN alignment strategy (Frazer et al., 2004) was applied to compare all the complete genomes with *A. argyrophylla* as reference. The contraction and expansion of the IR boundaries of five Rosaceae species were visualized using the IRscope software (Amiryousefi et al., 2018).

### Repeat Analysis and Codon Usage

REPuter online program (Kurtz et al., 2001) was used to identify long repeat sequences (forward, reverse, complementary, and palindromic). Repeat sequence locations and sizes in cp genomes were visualized with a minimal criterion of 30 bp, a hamming distance of 3, and less than 90% identity between two repeat copies. Tandem repeat finder (Benson, 1999) was used to identify tandem repeats in the two species *A. pedata* and *A. argyrophylla* cp genomes with default alignment parameters of match, mismatch, and insertions and deletions (indels) of 2, 7, and 7, respectively. SSRs were detected using the perl script MicroSAtellite (MISA) (Thiel et al., 2003) with a size motif of one to six nucleotides and a threshold of 10, 5, 5, 3, and 3 for mono-, di-, tri-, tetra-, penta-, and hexa-nucleotide, respectively. The codon usage frequency and relative synonymous codon usage (RSCU) of the two species were conducted based on 88 PCGs using MEGA 5 (Tamura et al., 2011).

### Adaptive Evolution and Substitution Rate Analysis

To evaluate the evolutionary rate variation within the *Alchemilla* species, 78 protein-coding regions within the cp genomes were explored with *F. virginiana* as reference species. PCGs were extracted using Geneious var. 9.1.4. Gaps and stop codons were manually removed, and the seven sequences were separately aligned using MAFFT v7. 308 (Katoh and Standley, 2013). The aligned files were converted into AXT format using the parseFastaIntoAXT.pl Perl script^2^. The non-synonymous (Ka) and synonymous (Ks) substitution rates as well as Ka/Ks ratio of each gene were estimated using the software KaKs_calculator 1.2 using the default model averaging (MA) method (Zhang et al., 2006). Taking into consideration that KaKs_calculator uses model averaging estimates in site selection, we implored the impact of site selection in 78 genes of five species phylogenetically related to *Alchemilla*. Positive selective pressure within shared genes of the seven species of subfamily Rosoideae was evaluated using PAML v4.7 (Yang, 2007) package implemented in EasyCodeML software (Gao et al., 2019). Non-synonymous (dN) and synonymous substitution (dS) substitution rates, and their ratio (ω = dN/dS) were calculated based on four site-specific models (M0 vs. M3, M1a vs. M2a, M7 vs. M8, and M8a vs. M8) with likelihood ratio test (LRT) threshold of *p* < 0.05 elucidating adaptation signatures within the genome. The models permit dN/dS variation within sites while keeping the ω ratio fixed within branches. Selective pressure analysis was conducted along ML tree in plain Newick format based on protein-coding sites used in the generation of phylogenetic relationship of the selected seven species. Here, individual coding DNA sequences (CDSs) were aligned in correspondence to their amino acids and their selection evaluated based on both ω and LRT values.

### Phylogenetic Analysis

Phylogenetic relationship analysis was done based on two data sets: (1) complete cp genome sequences (genomic tree) and (2) PCGs (CDS tree). The complete cp genomes of *A. pedata* and *A. argyrophylla* obtained from this study and other 24 cp genomes of Rosaceae species downloaded from NCBI database were inferred for the genomic tree (Supplementary Table S1). Multiple sequence alignment was performed using MAFFT v7.308 with default parameters setting. Phylogenetic relationship reconstructions were performed based on maximum-likelihood (ML) analysis using the program IQ-Tree v.6.1 (Nguyen et al., 2015) with 1,000 bootstrap replications. The best fit model TVM+I+G4 (Kalyaanamoorthy et al., 2017) was chosen according to Bayesian information criterion (BIC). The CDS tree was constructed by ML, PhyML, and BI methods based on 78 PCGs shared by all the 27 species under comparative evaluation. Gene sequences were extracted and aligned individually using Mega 7 and concatenated into a single file using PhyloSuite (Zhang et al., 2020). The ML CDS tree phylogenies were inferred using IQ-Tree with the best fit model GTR+F+R2 from ModelFinder in accordance to Akaike information criterion (AIC). Bayesian inference (BI) phylogenetic relationship of our taxa was constructed using MrBayes 3.2.6 (Ronquist et al., 2012) in PhyloSiute under GT+F+I+G4 best fit model from ModelFinder (two runs, 200,000 generations) following the AIC. The online program PhyMl vl 3.0 (Guindon et al., 2010) was used to infer phylogenetic relationship following GTR+G+I model selected by sms (Lefort et al., 2017). The constructed tree was visualized using FigTree version 1.4.4 (Rambaut, 2018).

## Results

### The Chloroplast Genome Structure and Content

**FIGURE 1:**
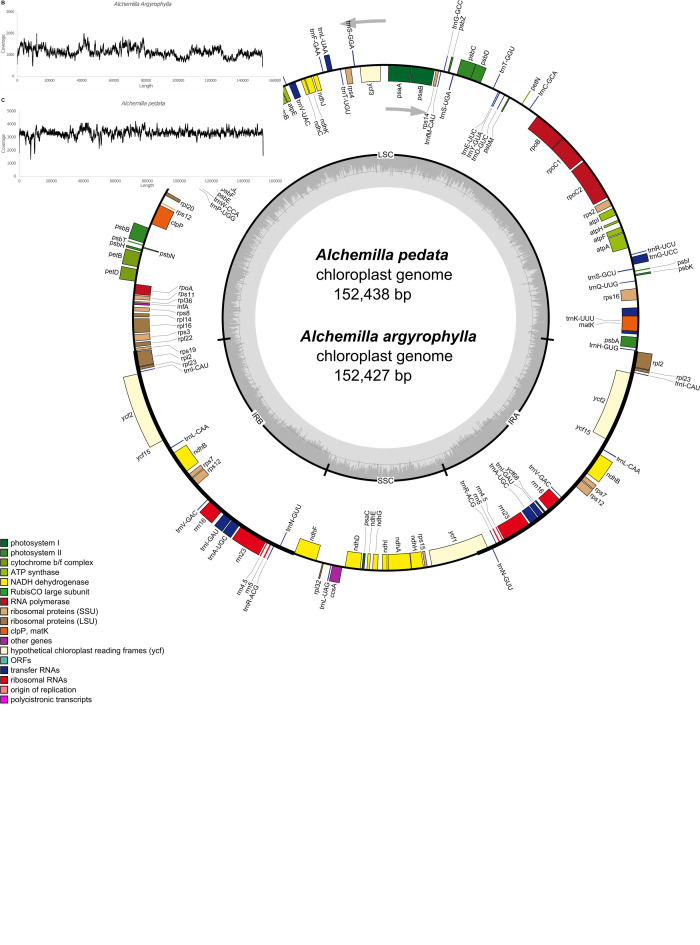
**(A)** The genome map of *Alchemilla pedata* and *Alchemilla argyrophylla* drawn using Organelle Genome DRAW software (Lohse et al., 2013). The genes outside the circle are transcribed in the counterclockwise direction, while those inside are transcribed in the clockwise direction. The colored bars indicate different functional groups. The lighter gray color denotes the AT content, while the gray area in the inner circle corresponds to the guanine–cytosine (GC) content of the genome. IRA, inverted repeat region A; IRB, inverted repeat region B; LSC, large singe copy; SSC, small single copy. **(B)** *A. argyrophylla* and **(C)** *A. pedata* graphs of read level coverage plots. The *de novo* assembly reads were trimmed, aligned, and mapped to their chloroplast genome after initial alignment with Bowtie in GetOrganelle using Geneious mapper (Kearse et al., 2012).

The total number of assembled reads was 3,383,185 and 1,158,253 with an average genome coverage depth of 3,299.6 and 1,133.5 in *Alchemilla pedata* and *Alchemilla argyrophylla*, respectively. The complete cp genomes display a typical circular structure with DNA sizes 152,438 and 152,427 bp for *A. pedata* and *A. argyrophylla*, respectively (Figure 1A). The quadripartite structure is composed of 82,612 bp in the LSC region, 17,980 bp in the SSC region, and the two IR regions made up of 25,923 bp each in *A. pedata*. *A. argyrophylla* has 82,616 bp in the LSC, 17,981 bp in the SSC region, and the two IR regions, each having 25,915 bp in length. The overall guanine–cytosine (GC) content for both *Alchemilla* species are 37% with 42.7, 34.9, and 30.4% in the IRs, LSC, and SSC, respectively. These are similar to other complete cp genome sequences of Rosaceae species obtained from NCBI database ranging between 36.6 and 37.1% (Table 1). To assess for any mis-assemblies, the raw reads were aligned against the *de novo* assembled genomes. The high per-base read coverages, plotted against the genomes position for each of the assemblies (Figures 1B,C), reveal the quality of our assembly. The annotated cp genomes were deposited in the GenBank database with the following accession numbers; *A. argyrophylla* MT382661 and *A. pedata* MT382662.

**TABLE 1:** Comparison of chloroplast genomes of 14 Rosaceae species.

Species	GenBank Acc. No.	Size (bp)	GC (%)	Protein	rRNA	tRNA	Gene
*Alchemilla argyrophylla*	MT382661	152,427	37	88	8	37	133
*Alchemilla pedata*	MT382662	152,438	37	88	8	37	133
*Dasiphora fruticosa*	MF683841	152,931	37.2	84	8	37	130
*Fragaria iinumae*	KC507759	155,554	37.1	85	8	37	130
*Fragaria nipponica*	KY769125	148,592	37.6	84	8	35	127
*Fragaria pentaphylla*	KY434061	155,640	37.3	85	8	35	129
*Fragaria orientalis*	KY769126	147,835	37.6	84	8	36	128
*Fragaria chiloensis*	JN884816	155,603	37.2	85	8	37	130
*Fragaria virginiana*	JN884817	155,621	37.1	85	8	37	130
*Fragaria mandshurica*	KC507760	155,596	37.2	85	8	37	130
*Fragaria vesca*	KC507760	155,691	37.2	85	8	37	130
*Rosa multiflora*	NC_039989	156,592	37.2	90	8	37	136
*Rosa odorata* var. *gigantea*	KF753637	156,634	37.2	88	8	40	139
*Hagenia abyssinica*	KX008604	154,961	37.1	85	8	37	129

*GC, guanine–cytosine; rRNA, ribosomal RNA; tRNA, transfer RNA.*

A total of 114 unique genes were annotated including 88 PCGs, 37 transfer RNA (tRNA) genes, and 4 ribosomal RNA (rRNA) genes in both *A. argyrophylla* and *A. pedata* (Table 1). Based on their gene functional category, 59 genes were associated with self-replication while 44 genes are responsible for photosynthesis (Table 2). Similar gene order and genome structure were reported in both *Alchemilla* species (Figure 1A). The IR regions (IRa and IRb) had 18 duplicate genes comprising seven PCGs (*rpl2*, *rpl23*, *ycf2*, *ycf15*, *ndhB*, *rps7*, and *rps12*), seven tRNA (*trnI-CAU*, *trnL-CAA*, *trnV-GAC*, *trnI-GAC*, *trnA-UGC*, *trnR-ACG*, and *trnN-GUU*), and four rRNAs (*rrn16*, *rrn23*, *rrn4.5*, and *rrn5*). The SSC region had 13 genes of which 12 were PCGs and one tRNA, whereas the LSC had 62 PCGs and 22 tRNA (Figure 1A). In total, 15 genes (*trnK-UUU*, *rps16*, *trnG-UCC*, *rpoC1*, *trnL-AUU*, *trnV-UAC*, *petB*, *petD*, *rpl16*, *rpl2*, *ndhB*, *trnA-UGC*, *ndhA*, *trnA-UGC*, and *trnI-GAU*) had one intron with *rpl2* and *ndhB* duplicated in the IR, whereas two genes, *clpP* and *ycf3*, had two introns. The *rps12* is a trans-spliced gene, with one exon shared between two introns, in which the 3′ exons were duplicated in the IR regions and the 5′ exon end situated in the LSC region (Table 2). Among the 133 genes, three instances of overlapping sequences were detected in *Alchemilla.* The *psbD* and *psbC* genes shared coding regions (53 bp); *ycf68* gene was embedded within *trnI-GAU* in one of the inverted region (IRa); and *matK*, which has the longest intron (2,523 bp in *A. argyrophylla* and 2,528 bp in *A. pedata*), was embedded in *trnK-UUU* in the SSC region (Figure 1A).

**TABLE 2:** Genes present and functional gene category in *Alchemilla pedata* and *Alchemilla argyrophylla* chloroplast genome.

Functional category	Group of genes	Name of genes
Transcription and translation related genes (self-replication)	DNA-dependent RNA polymerase	*rpoA*, *rpoB*, *rpoC1, *rpoC2*
	Ribosomal proteins (SSU)	*rps2*, *rps3*, *rps4*, ***rps7***, *rps8*, *rps11*, *rps12, *rps14*, *rps15*, *rps16, *rps18*, *rps19*
	Ribosomal protein (LSU)	*rpl2, *rpl14*, *rpl16, *rpl20*, *rpl22*, ***rpl23***, *rpl33*, *rpl32*, *rpl36*
RNA genes (self-replication)	Ribosomal RNA	***rrn4.5***, ***rrn5***, ***rrn16***, ***rrn23***
	Transfer RNA	*trnA-UGC, *trnC-GCA*, *trnD-GUC*, *trnE-UUC*, *trnF-GAA*, *trnfM-CAU*, *trnG-GCC*, *trnG-UCC, *trnH-GUG*, *trnI-CAU*, *trnI-GAU, *trnK-UUU, ***trnL-CAA***, *trnL-UAA, *trnL-UAG*, *trnM-CAU*, ***trnN-GUU***, *trnP-UGG*, *trnQ-UUG*, ***trnR-ACG***, *trnR-UCU*, *trnS-GCU*, *trnS-GGA*, *trnS-UGA*, *trnT-GGU*, *trnT-UGU*, ***trnV-GAC***, *trnV-UAC, *trnW-CCA*, *trnY-GUA*
Photosynthesis related	Rubisco	*rbcL*
	Photosystem I	*psaA*, *psaB*, *psaC*, *psaI*, *psaJ*
	Photosystem II	*psbA*, *psbB*, *psbC*, *psbD*, *psbE*, *psbF*, *psbH*, *psbI*, *psbJ*, *psbK*, *psbL*, *psbM*, *psbN*, *psbT*, *psbZ*
	ATP synthase	*atpA*, *atpB*, *atpE*, *atpF*, *atpH*, *atpI*
	Cytochrome b/f complex	*petA*, *petB, *petD, *petG*, *petL*, *petN*
	NADPH dehydrogenase	*ndhA, *ndhB, *ndhC*, *ndhD*, *ndhE*, *ndhF*, *ndhG*, *ndhH*, *ndhI*, *ndhJ*, *ndhK*
Other genes	Maturase	*matK*
	cytochrome *c*-type synthesis	*ccsA*
	Carbon metabolism	*cemA*
	Fatty acid synthesis	*accD*
	Transfer initiation factor	*infA*
	Proteolysis	**clpP
Genes of unknown function/Hypothetical Protein RF	Conserved open reading frames	*ycf1*, ***ycf2***, **ycf3, *ycf4*, ***ycf68*, *ycf15***

Genes in bold are located within the inverted repeat (IR) and therefore in duplicates except ycf68. *Genes have one intron. **Genes have two introns.

### Codon Usage Analysis

The CDSs of the cp genomes were used to estimate the frequency of codon usage of both *A. pedata* and *A. argyrophylla*. A total of 22,948 and 22,984 codons encoding 88 genes were detected in *A. argyrophylla* and *A. pedata*, respectively. Of all the codons, leucine reported the highest amino acid usage frequency of 10.57% (2,426) in *A. argyrophylla* and 10.58% (2,432) in *A. pedata*, whereas cysteine had the lowest amino acid usage frequency of 1.09% (251) in *A. argyrophylla* and 1.08% (249) in *A. pedata* (Supplementary Figure S1). The most frequently used codons are AUU (993 and 996), AAA (938 and 940), and GAA (922 and 921) encoding isoleucine, lysine, and glutamic acid in *A. argyrophylla* and *A. pedata*, respectively (Supplementary Table S2). Furthermore, RSCU value was estimated in the 88 CDSs in both *Alchemilla* species. RSCU is the ratio between the expected frequency of use and the actual frequency usage of a particular codon. Codons reporting RSCU value < 1 indicates lower frequency usage than expected, while a score > 1 signifies higher usage frequency (Sharp and Li, 1987; Munyao et al., 2020). In *Alchemilla* species, apart from the stop codon (UGA), isoleucine (I) codon AUA and leucine (L) codon (CUA) with RSCU value below 1, all the other codons with synonymous codons usage (RSCU > 1) preferred to end with A or U in both *A. pedata* and *A. argyrophylla* signifying their preferential codon use. Exceptionally, codon UUG encoding leucine recorded higher bias of RSCU = 1.19 in both species despite ending with G in the third position than did other codons of low frequencies (RSCU < 1) that ended in C or G. Codons AUG (M) and UGG (W) encoding methionine and tryptophan showed no bias (RSCU = 1) (Supplementary Table S2). Our findings are consistent with majority cp genomes of land plants (Cheng et al., 2017). Due to usage frequency variation, RSCU values of the cp genome form a valuable source of evolutionary signature traits resulting from mutation and selection that are essential in studying the evolution of an organism (Morton, 2003; Wang et al., 2018).

### Repeat Structure and Simple Sequence Repeats

**FIGURE 2:**
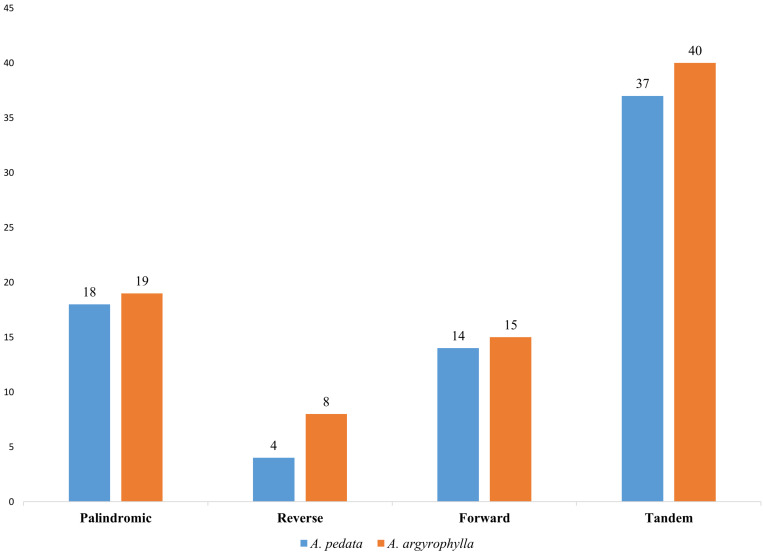
Number of long repeat sequence types in *Alchemilla pedata* and *Alchemilla argyrophylla* in the distribution of palindromic, reverse, forward, and tandem repeats.

Repeat motifs are significant in the computation of phylogenetic and genomic rearrangement (Cavalier-Smith, 2002). In this report, *A. pedata* reported 36 long repeats comprising four palindromic (P), 14 reverse (R), and 18 forward (F) repeats, whereas *A. argyrophylla* recorded 42 long repeats composed of 19 P, 8 R, and 15 F repeats (Figure 2). In both species, complementary repeats were not found. This is similar to finding obtained in other Rosaceae species (Gichira et al., 2017). The comparative analysis results revealed that most repeats were between 30 and 40 bp. The longest repeat was a palindromic repeat having 71 bp located in the intergenic spacer (IGS) of the LSC region between *trnM-CAU* and *atpE* in both species (Supplementary Table S3). Most repeats were distributed in LSC (non-coding) region, whereas some were found in genes including *ndhA*, *ycf3*, *ycf1*, *rpoC1*, *rpl16*, and *ndhB.* Six repeats were found exclusively in the IR regions, three of them relating to *ndhB* gene (Supplementary Table S3). The number of tandem repeats was 40 in *A. argyrophylla* and 37 repeats in *A. pedata* (Figure 2). In *A. argyrophylla*, only four repeats were > 30 bp in length, while the rest were between 1 and 28 bp. Of these repeat units, 22 repeats had mismatches, and 15 had indels (Supplementary Table S4a). In *A. pedata*, two repeats were > 30 bp, and the rest were between 9 and 28 bp in length. Sixteen repeat units reported mismatches, and 11 had indels (Supplementary Table S4b).

**FIGURE 3:**
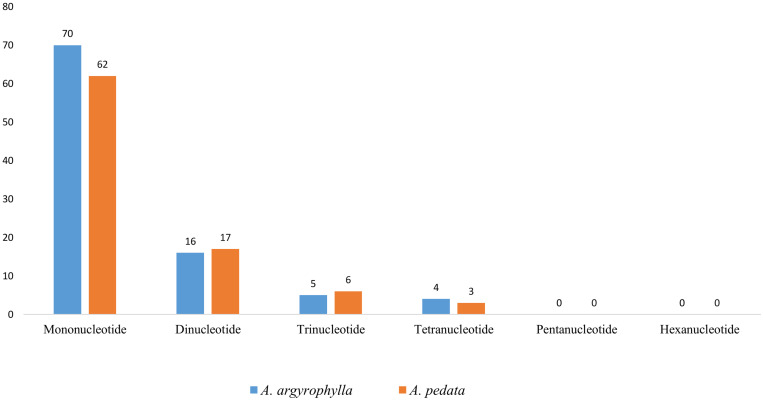
Number of different simple sequence repeat (SSR) units in *Alchemilla argyrophylla* and *Alchemilla pedata* comprising mono-, di-, tri, tetra-, penta-, and hexa-nucleotide repeats.

SSRs, also called short tandem repeats or microsatellites, are repeating sequences of about 1–6 bp that are uniparentally inherited and widely distributed in the whole cp genome (Cheng et al., 2015). SSRs are ideally co-dominant, having the highest degree of intraspecific polymorphism (Weber, 1990), high mutation rates, locus specificity, and multi-allelism (Kuang et al., 2011; Asaf et al., 2017). Thus, the microsatellites are valuable markers ideal for molecular breeding (Rafalski and Tingey, 1993), population genetics (Powell et al., 1995), gene mapping, and genetic linkage analysis (Pugh et al., 2004; Xue et al., 2012). In our study, a total 95 SSRs were identified in *A. argyrophylla* composed of 70 mononucleotides, 16 dinucleotides, 5 trinucleotides, and four tetranucleotides (Figure 3 and Table 3). Similarly, *A. pedata* cp genome had 88 SSRs composed of 62 mononucleotides, 17 dinucleotides, 8 trinucleotides, and 3 tetranucleotides (Figure 3 and Table 3). In both species, mononucleotides were the most abundant repeat types (*A. argyrophylla* 73.68% and *A. pedata* 70.45%). Pentanucleotides and hexanucleotides were not detected in both species (Figure 3). Apart from one mononucleotide, all the other SSRs were rich in A and T (Table 3). These findings are consistent with contention that SSRs are typically composed of polyadenine (PolyA) and polythyamine (PolyT) repeats in line with previous reports (Cheng et al., 2015; Shen et al., 2016). This perpetually contributes to biasness in base composition of the whole cp genome, where A/T content in the reported *Alchemilla* species is 62.98% in *A. argyrophylla* and 62.99% in *A. pedata* compared with the GC content represented by 37.0% in both species.

**TABLE 3:** Number of different simple sequence repeats (SSRs) in *Alchemilla argyrophylla* and *Alchemilla pedata.*

SSRs	*A. argyrophylla*	*A. pedata*
A/T	69	61
C/G	1	1
AT/TA	16	17
AAT/ATT	5	6
AAAT/ATTT	3	2
AATT/AATT	1	1
Total no. of SSRs	95	88

*SSRs, simple sequence repeats.*

### Comparative Genome Analysis

For further analysis of the cp genome of *Alchemilla* species, 12 whole cp genome sequences of the Rosaceae species were downloaded from NCBI, and the basic genomic characteristics were compared (Table 1). A high similarity was observed in all genome sequences. The genome size ranged from 148,592 bp in *Fragaria nipponica* to 156,634 in *Rosa odorata* var. *gigantea* and was concurrent with the lowest and highest number of genes that ranged from 127 to 139, respectively. The number of PCGs ranged from 84 in *Fragaria orientalis* to 90 in *Rosa multiflora*, whereas tRNA genes ranged from 35 in *F. nipponica* to *Fragaria pentaphylla* to 40 in *R. odorata* var. *gigantea.* The cp genome structure and gene arrangement are conserved in all the species evaluated (Table 1).

To ascertain divergence within the cp genome, multiple alignment analysis of *Alchemilla* species and seven other Rosacea cp genomes were conducted using mVISTA program. Results showed that IR regions have higher similarity than the SC regions (Supplementary Figure S2). Higher conservation was observed in the coding regions than the non-coding regions, which is a common phenomenon in most angiosperms (Cheng et al., 2017). Significantly, the most conserved regions were observed in the tRNA and rRNA regions across all species. High variation was observed in the IGS regions of *trnH-GUG*–*psbA*, *trnK-UUU*–*rps16*, *rps16*–*trnQ-UUG*, *trnR-UCU*–*atpA*, *ndhC*–*trnV-UAC*, *atpF*–*atpH*, *trnC-GCA*–*petN*, *petN*–*psbM*, *trnD-GUC*–*trnY-GUA*, *psaA*–*ycf3*, *rps4*–*trnT-UGU*, *trnL-UAA*–*trnF-GAA*, *ndhC*–*trnV-UAC*, *petD*–*rpoA*, *rps11*–*rpl36*, *trnL-CAA*–*ndhB*, and *PetA*–*psbJ* in the LSC. Non-coding regions in *ndhF*–*rpl32*, *rpl32*–*trnL-UAG*, *ccsA*–*ndhD*, and *rps15*–*ycf1* reported high divergence in the SSC. Coding regions with the highest variation include *ycf1*, *ndhF*, *rpoA*, *infA*, *accD*, *rpoC2*, and *matK*. Divergence was also detected in introns of *trnK-UUU*, *rps16*, *ycf3*, *petD*, *rpl16*, *clpP*, *rps12*, and *ndhA.* These are regions of rapid evolutionary changes and therefore are essential sites for the development of molecular markers that could be useful in population genetics and phylogenetic studies. Our results are consistent with findings of other Rosaceae species (Shen et al., 2016; Jian et al., 2018). Generally, *A. pedata* and *A. argyrophylla* are highly similar to *Dasiphora* and *Fragaria* species and most divergent from *Pentactina* and *Prinsepia* species among the seven evaluated species (Supplementary Figure S2).

### Expansion and Contraction of the Inverted Repeat Regions

**FIGURE 4:**
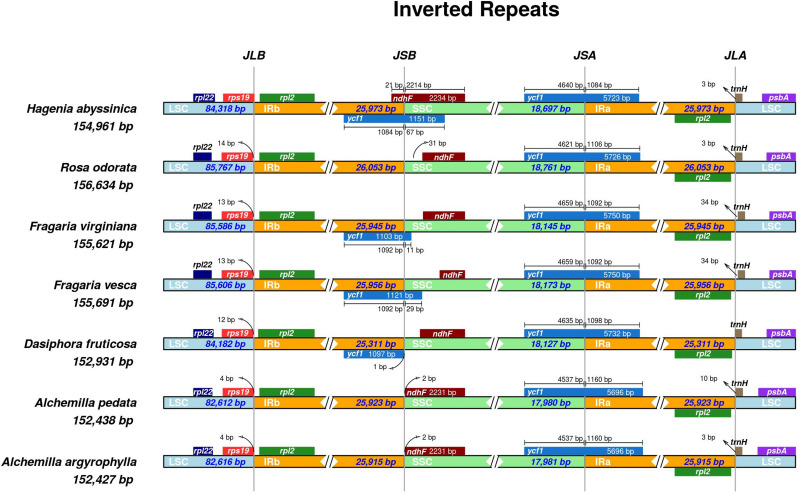
Comparative distance between the boundaries of the two inverted repeats (IRa/IRb), small single copy (SSC), and large single copy (LSC) regions and adjacent genes among the chloroplast genomes of seven Rosaceae species using IRscope software (Amiryousefi et al., 2018). The species name and their corresponding genome lengths are shown on the left side of the figure. JLB, JSB, JSA, and JLA correspond to LSC/IRb, IRb/SSC, SSC/IRa, and IRa/LSC junctions, respectively. Genes drawn above the track indicate direct transcription, and genes below the track indicate complement transcription. The arrows indicate the distance from the beginning or ending coordinate of the specific gene from the corresponding junction; AT bar above or below the gene extending into two regions shows to what extent in base pairs it has stretched. The figure is not drawn to scale based on sequence length but only shows the relative change near or at the IR/SC junctions.

The IR boundaries of *A. pedata* and *A. argyrophylla* were compared with those of five other species of Rosaceae to analyze probable expansion or contraction in the IR (Figure 4). Despite the IRs being the most conserved region of the cp genome, constant variation in the position of the IR/SC boundary and their associated adjacent genes observed in plant lineages has been because of the contraction and expansion of the IR region, which subsequently acts as an evolutionary indicator (Wang et al., 2008). Our results showed that the different species had varied IR sizes, ranging from 25,311 bp in *Dasiphora fruticosa* to 26,053 bp in *R. odorata* (Figure 4). The *rpl22* and *rps19* genes lied exclusively in the LSC region adjacent to the LSC/IRb junction, while the *rpl19* shifted away from the LSC/IRb boundary with gap of 4–14 bp. The *ndhF* gene in the analyzed species was located entirely in the SSC region having varied distances from the IRa/SSC boarder (JBL). However, *Hagenia abyssinica* had the *ndhF* gene stretching 21 bp into IRb region. The *ycf1* gene stretched through the SSC/IRa boarder (JSA) in all the species at varied lengths. The *trnH* gene located entirely in the LSC region stretched 3–34 bp away from the IRa/LSC junction in all the analyzed species. Generally, the *trnH* gene in monocots is located in the IR region, while that in dicots is located in the LSC region (Asano, 2004; Cheng et al., 2017).

### Adaptive Evaluation Analysis

Non-synonymous (Ka) and synonymous (Ks) substitutions and their proportional ratios (Ka/Ks) similarly referred to as (dN/dS) have been used to evaluate nucleotide’s natural selection pressure and evolution rates (Ninio, 1984; Yang and Nielsen, 2000). In most protein-coding regions, occurrence of synonymous substitutions has been reported more frequently than occurrence of non-synonymous substitutions (Makałowski and Boguski, 1998). The synonymous substitutions normally do not alter the amino acid chain unlike the non-synonymous substitutions that change the amino acid sequence. In this study, Ka and Ks values were estimated in 78 genes of the *A. pedata* and *A. argyrophylla*, computed against a close relative *Fragaria virginiana* (Supplementary Table S5). In our evaluation, none of the genes reported Ka value above 1 of which *ycf1* PCG reported the highest value (Ka = 0.0416) in *A. pedata* and (Ka = 0.0411) in *A. argyrophylla*. On the other hand, the highest Ks value was recorded in photosynthesis gene *psbD* (Ks = 1.2010) in *A. pedata* and *petL* (Ks = 0.2642) in *A. argyrophylla*.

The Ka/Ks value indicates the intensity of selective pressure imposed on a particular gene. Neutral selection is denoted by a Ka/Ks value of 1, Ka/Ks ratio < 1 signifies negative (purifying) selection, and Ka/Ks ratio > 1 indicates positive (adaptive) selection (Nei and Kumar, 2000). Purifying selection is common in many protein-coding regions (Nielsen, 2005). In this study, most of the genes had Ka/Ks ratio of less than 0.5, accounting for over 90% of the analyzed genes. However, the high Ka/Ks values were noted in *rps7* (Ka/Ks = 50), *rpl23* (Ka/Ks = 50), and *psbJ* (Ka/Ks = 47) in *A. argyrophylla* and *rpl32* (Ka/Ks = 50) and *psbJ* (Ka/Ks = 47) in *A. pedata* due to very low Ks value < 0.001 implying 0 synonymous changes in the genes. This means that there was very low or no substitutions (NA) between the aligned gene sequences (Mo et al., 2020). We therefore replaced the high Ka/Ks values in these genes with 0. The average Ka/Ks value was found to be 0.1322 in *A. argyrophylla* and 0.1418 in *A. pedata*, signifying an overall negative selection pressure of the genes (Supplementary Table S5). Genes with Ka/Ks > 0.5 included *petN*, *psbL*, and *psbN* in *A. argyrophylla* and *petN*, *psbD*, *psbL*, and *PsbN in A. pedata*. In both *Alchemilla* species, the least Ka/Ks value (0.0010) was recorded in photosynthesis-related genes (*atpH*, *ndhI*, *petD*, *petD*, *petG*, *petL*, *psaC*, *psbA*, *psbH*, *psbI*, *psbM*, and *psbT*) and self-replicating genes (*rps2*, *rps19*, and *rps36*), indicating significant purifying selection (Supplementary Table S5). The same functional protein-coding sequences in seven Rosoideae species were used to detect sites of positive selection. Among the four models, comparative LRT of M7 vs. M8 was positive in determining *p*-value of chi square < 0.05 and the selection strength. Bayes empirical Bayes (BEB) (Yang et al., 2005) and naïve empirical Bayes (NEB) analyses were implemented in model M8. In the BEB method, three sites were detected as site of positive selection, which represented one photosynthesis-related gene *ndhB*, self-replication gene *rpoC1*, and hypothetical gene *ycf2* (Table 4). NEB method on the other hand detected 59 sites that coded for 17 genes under selective pressure. Among the genes, *rpoc2*, *ycf2*, and *ndhB* had *p* > 0.99%. The generally slow evolutionary rates and subsequent low Ka/Ks ratio observed in *Alchemilla* species is a common attribute of the cp genome. The varying results of Ka/Ks ratio obtained in our study give evidence that evolutionary rates of cp genomes vary among genes. Similar conclusions were drawn by Menezes et al. (2018) in the cp genome analysis of Malpighiales.

**TABLE 4:** Positively selected sites detected in the chloroplast genome of subfamily Rosoideae based of Bayes empirical Bayes (BEB) method.

	M8	
Gene name	Selected sites	Pr (w > 1)
*rpoC1*	3,755 P	0.994**
*ycf2*	5,727 I	0.983*
*ndhB*	13,616 I	0.964*

* p < 0.05.** p < 0.01.

### Phylogenetic Analysis

**FIGURE 5:**
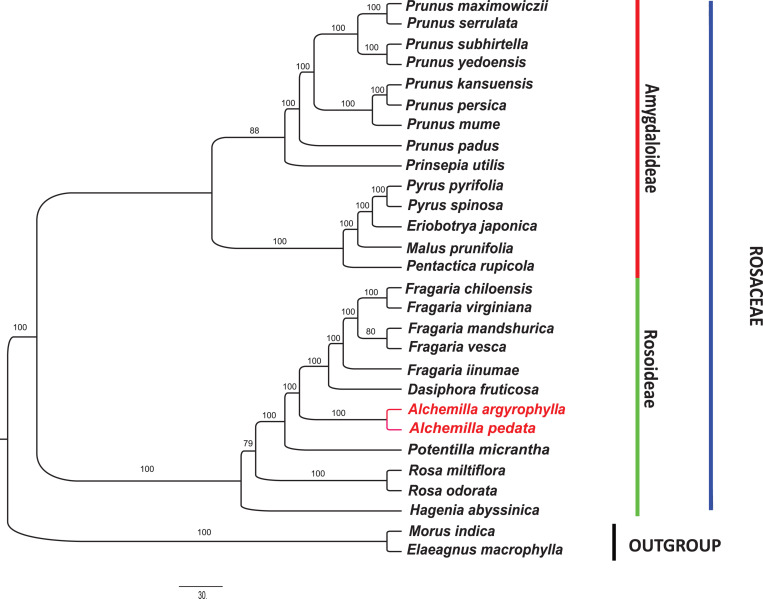
Phylogenetic tree reconstruction of Rosaceae species based on maximum-likelihood (ML) analysis using the program IQ-Tree v.6.1 (Nguyen et al., 2015) with 1,000 bootstrap replications in 26 complete chloroplast genome sequences. *Morus indica* and *Elaeagnus macrophylla* were used as outgroups.

**FIGURE 6:**
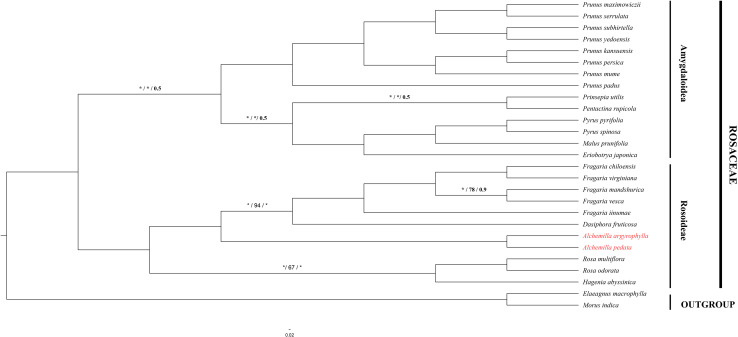
Phylogenetic tree reconstruction based on maximum-likelihood IQ-Tree in PhyloSuite (Zhang et al., 2020) with 1,000 replications, PhyML (Lefort et al., 2017) and BI based on MrBayers (Ronquist et al., 2012) in 200,000 generation using 78 protein-coding genes common in 27 Rosaceae species. The numbers above the branch represent bootstrap support value for ML/PhyML/BI methods, where the asterisk signifies maximum support value of 100 in IQ and 1 BI. Blank branches signify 100% support value.

In order to understand the evolutionary relationship among Rosaceae species, complete cp genome sequences and 78 PCGs in 27 species of subfamily Rosoideae and Amygdaloideae were used to infer the phylogenetic position of *A. argyrophylla* and *A. pedata* with *Elaeagnus macrophylla* (Elaeagnaceae) and *Morus indica* (Moraceae) as outgroups. The cp genome sequences and its PCGs provide precise and systematic genomic information for phylogenetic and evolutionary relationship reconstruction (Yang X. et al., 2020). Phylogenetic analysis was conducted using ML, BI, and PhyML methods (Figures 5,6). A comparison between the CDS tree and the genome tree revealed an overall similar topology with few incongruences observed in subfamily Amygdaloideae (Figure 6). In both trees, *Alchemilla* species were found to be closely related to species of genus *Fragaria* and *Dasiphora* with strong bootstrap support in the subfamily Rosoideae. The *Alchemilla* species clustered together, assuming a monophyletic clade. Our findings are consistent with previous phylogenies reconstructed including representatives of the genus *Alchemilla* using molecular markers (Eriksson et al., 2003; Xiang et al., 2016; Zhang et al., 2017).

## Discussion

### *Alchemilla* Chloroplast Genome, Conserved Genome Structure, and Gene Content

Our study is the initial report and analysis of the complete cp genome of the *Alchemilla* species. They are represented by *Alchemilla pedata* and *Alchemilla argyrophylla*, a herb and a shrub, respectively, of the *Afromilla* clade found in the cooler mountainous regions of East Africa at altitudes of 2,250–4,500 m above sea level (Graham, 1960). The sequenced cp genome comprises 114 unique genes with slight variation in genome size of 152,427 bp in *A. argyrophylla* and 152, 438 bp in *A. pedata* (Figure 1A). Compared with other species, the size variation could be a result of the expansion and contraction of the IR region (Palmer et al., 1988). Similar to other cp genomes of higher plants, the genome annotation of *Alchemilla* species revealed an LSC-IR–SSC-IR arrangement as well as a systemic gene category and functional classification. Comparative analysis with other Rosaceae species reveals the conserved structural and organizational nature with slight variation in gene content and genome length (Table 1). Furthermore, the hypothetical *ycf68* embedded within *trnI-GUA*, previously not annotated in the most Rosacea species, was detected in the two annotated *Alchemilla* species (Figure 1A). Nucleotide substitution rates in the cp genome of angiosperms are considered lower than those of the nuclear genome (Wolfe et al., 1987). Therefore, the low gene substitution rate reported by the PCGs in *Alchemilla* is consistent with other cp genomes of higher plants. Concomitantly, the low rate of nucleotide substitutions in the PCG could be accentuated by recombination between the IRs. This is primarily due to the recurrent intra-chromosomal recombination event interplay between the two identical IR regions of the cp genome. As a result, selective constraints are imposed on both the structural stability and the sequence homogeneity (Wolfe et al., 1987; Palmer et al., 1988). The ratios between non-synonymous and synonymous substitutions (Ka/Ks) are fundamental in elucidating natural selection pressure (Nei and Kumar, 2000). In *Alchemilla*, *petN*, *psbL*, and *psbN* with Ka/Ks > 0.5 and *rpoC2*, *ycf2*, and *ndhB* with *p* > 0.99% are essential in unfolding evolutionary history of the genus (Table 4 and Supplementary Table S5). The genomic information in this study will be fundamental in the phylogenetic studies as well as the generation of molecular markers of not only the African *Alchemilla* clade but also genus *Alchemilla*.

### The Loss of *atpF* Group II Intron

Introns are generally conserved regions among land plants; and therefore, instances of intron loss or gain in the cp genome could signify an evolutionary event (Daniell et al., 2016). In the annotated *Alchemilla* cp genomes, we report the absence of an intron in *atpF* belonging to group II introns. This is a rare phenomenon in land plant besides Euphorbiaceae and Malpighiales (Daniell et al., 2008). A similar observation was made in *Fragaria vesca* for the first time in Rosoideae (Shulaev et al., 2011), which we found to be closely related to our species based on phylogenetic analysis (Figure 5). Subsequent absence of the *atpF* intron has been evident in species of *Rosa*, *Potentilla*, *Rubus*, and *Fragaria* (Yang J. et al., 2020). However, a comparison with relative species of subfamily Amygdaloidea indicates the retention of introns within *atpF* genes (Supplementary Table S6). Based on the present phylogenetic framework, the loss of intron within *atpF* genes seems to have taken place once within the Rosoideae subfamily. The loss of intron in *atpF* genes is yet to be determined in other species of subfamily Rosoideae, genera in the Rosaceae, and the broader families of Rosids (Yang J. et al., 2020). Introns are broadly classified as either group I or group II and are considered as the mobile genetic elements in the cp genome. The cp genome of land plants has 17 to 20 introns classified under group II within tRNA and PCGs (Daniell et al., 2008). The only group I intron present within *trnL-AUU* gene is considered most ancient since it is also found in cyanobacteria other than in land plants and algae’s cp genome (Vogel et al., 1999). The splicing function of *trnL* and other RNA transcripts for genes such as *trnA*, *rpl2*, *rps12*, and *atpF* is fully dependent on the function of maturases. The *matK* is the only maturase encoded protein present in the cp genome of land plants, yet it lacks the reverse transcriptase domain and hence cannot promote intron mobility (Hao et al., 2010; Liu et al., 2016). This causes the splicing of group II introns, including *atpF* intron, to be dependent on the host encoded splicing factors through lariat formation (Barkan, 2004). The loss of *atpF* intron among *Alchemilla* species and its close relatives could be used as a new classification basis of structural change within Rosaceae. Furthermore, the presence or absence of introns maybe insightful in understanding phylogenetic relationships as well as providing a potentially resourceful marker for evolutionary lineages in angiosperms.

### Phylogenetic Analysis of *Alchemilla*

Over the previous century, the taxonomic treatment of Rosaceae has yielded varied results pertaining to its family position among the angiosperms (Potter et al., 2007). Furthermore, classification within the family has always differed depending on the treatment imposed (Hutchinson, 1964; Schulze-Menz, 1964; Kalkman, 2004; Potter et al., 2007). Morgan et al. (1994) resolved that Rosaceae be composed of monophyletic groups based on phylogenetic analysis of *rbcL* sequences across the family. This acclamation was later supported by phylogenetic studies based on *ndhF*, *matK*, and *trnL-trnF* sequences (Potter et al., 2002; Zhang et al., 2017). However, there exist uncertainties within genera resulting from unresolved tree portions having weak support (Potter et al., 2007). Notably in the genus *Alchemilla*, the distinction between the African clade (*Afromilla*) and the Eurasian (*Eualchemilla*) clade is geographically significant but remain unresolved (Gehrke et al., 2016). The contention requires the separation of the *Alchemilla* into *Eualchemilla* and *Afromilla* clade, which has been restrained due to lack of substantial morphological borderlines despite extensive and significant studies (Notov and Kusnetzova, 2004; Gehrke et al., 2008; Lundberg et al., 2009). The emergence and rapid development in cp genome sequencing technologies have been essential in providing resourceful genomic information that has been fundamental for the reconstruction of lower and higher plant phylogenies as well as evolutionary trends (Daniell et al., 2016; Yang X. et al., 2020). Significantly, the cp genome sequences have been proven effective in eliminating phylogenetic incongruences arising from incomplete lineage sorting (ILS) and hybridization (Lundberg et al., 2009; Morales-Briones et al., 2018). In this study, *A. pedata* and *A. argyrophylla* nested into a monophyletic clade with 100% bootstrap support. They share a recent ancestry with *Dasiphora* and *Fragaria* species (Figure 5). There is need for more complete cp genomes of *Alchemilla* species for higher precision in phylogenetic conclusion. This study is therefore resourceful for further delimitation of species in *Alchemilla* and phylogenetic studies of the genus.

## Data Availability Statement

The assembled chloroplast genome sequences have been uploaded to and deposited in GenBank and we have received the accession numbers which are shown in Table 1 in the manuscript.

## Author Contributions

PR and XD performed the experiment. PR, XD, and J-XY performed the data analysis. PR drafted the manuscript. FM, MO, IM, and PK revised the manuscript. G-WH and Q-FW designed and supervised the experiment. All the authors contributed to and approved the final manuscript.

## Conflict of Interest

The authors declare that the research was conducted in the absence of any commercial or financial relationships that could be construed as a potential conflict of interest.

## Footnotes

1 https://www.ncbi.nlm.nih.gov/

2 http://code.google.com/p/kaks-calculator/
