# Supplementary material for: Initial Complete Chloroplast Genomes of Alchemilla (Rosaceae): Comparative Analysis and Phylogenetic Relationships
Source: Front Genet. 2020 Dec 9;11:560368. doi: 10.3389/fgene.2020.560368 (PMC7756076; doi:10.3389/fgene.2020.560368)
Supplement: Supplementary file 1 [file Table_1.DOCX]

Supplementary Material

# Supplementary Tables

**Supplementary Table S1**. Complete chloroplast genome accession numbers of 24 Rosacea species downloaded from NCBI database

| Species name | Accession no. |
| --- | --- |
| *Prunus maximowiczii* | KP760071 |
| *Prunus serrulata* var. *spontanea* | KP760073 |
| *Prunus subhirtella* var. *subhirtella* | KP760075 |
| *Prunus yedoensis* | KP760070 |
| *Prunus kansuensis* | KF990036 |
| *Prunus persica* | HQ336405 |
| *Prunus mume* | KF765450 |
| *Prunus padus* | KP760072 |
| *Prinsepia utilis* | KC571835 |
| *Pyrus pyrifolia* | AP012207 |
| *Pyrus spinosa* | HG737342 |
| *Eriobotrya japonica* | NC_034639 |
| *Malus prunifolia* | KU851961 |
| *Pentactina rupicola* | JQ041763 |
| *Fragaria chiloensis* | JN884816 |
| *Fragaria virginiana* | JN884817 |
| *Fragaria mandshurica* | KC507760 |
| *Fragaria vesca* | JF345175 |
| *Fragaria iinumae* | KC507759 |
| *Dasiphora fruticosa* | NC_036423 |
| *Potentilla micrantha* | HG931056 |
| *Rosa multiflora* | NC_039989 |
| *Rosa odorata* var. *gigantea* | KF753637 |
| *Hagenia abyssinica* | KX008604 |

**Supplementary Table S2.** Amino acids, Relative synonymous Codon usage (RSCU) and percentage (%) RSCU analysis of *Alchemilla argyrophylla* and *Alchemilla pedata*

| Amino Acid | Codon | Count (*A. argyrophylla*) | RSCU | % RSCU | Count (*A. pedata*) | RSCU | % RSCU |
| --- | --- | --- | --- | --- | --- | --- | --- |
| Phenylalanine(F) | UUU | 904 | 1.38 | 5.712916 | 908 | 1.38 | 5.730073 |
|  | UUC | 407 | 0.62 |  | 409 | 0.62 |  |
| Leucine(L) | UUA | 830 | 2.05 | 10.57173 | 834 | 2.06 | 10.58127 |
|  | UUG | 483 | 1.19 |  | 483 | 1.19 |  |
|  | CUU | 506 | 1.25 |  | 509 | 1.26 |  |
|  | CUC | 146 | 0.36 |  | 144 | 0.36 |  |
|  | CUA | 310 | 0.77 |  | 309 | 0.76 |  |
|  | CUG | 151 | 0.37 |  | 153 | 0.38 |  |
| Isoleucine(I) | AUU | 993 | 1.51 | 8.602057 | 996 | 1.51 | 8.614688 |
|  | AUC | 337 | 0.51 |  | 335 | 0.51 |  |
|  | AUA | 644 | 0.98 |  | 649 | 0.98 |  |
| Methionine(M) | AUG | 526 | 1 | 2.292139 | 526 | 1 | 2.288549 |
| Valine(V) | GUU | 473 | 1.47 | 5.608332 | 477 | 1.48 | 5.6126 |
|  | GUC | 139 | 0.43 |  | 138 | 0.43 |  |
|  | GUA | 505 | 1.57 |  | 508 | 1.58 |  |
|  | GUG | 170 | 0.53 |  | 167 | 0.52 |  |
| Serine(S) | UCU | 477 | 1.69 | 7.386265 | 476 | 1.68 | 7.383397 |
|  | UCC | 250 | 0.88 |  | 250 | 0.88 |  |
|  | UCA | 323 | 1.14 |  | 325 | 1.15 |  |
|  | UCG | 176 | 0.62 |  | 174 | 0.62 |  |
|  | AGU | 356 | 1.26 |  | 359 | 1.27 |  |
|  | AGC | 113 | 0.4 |  | 113 | 0.4 |  |
| Proline(P) | CCU | 359 | 1.5 | 4.183371 | 361 | 1.5 | 4.176819 |
|  | CCC | 191 | 0.8 |  | 192 | 0.8 |  |
|  | CCA | 253 | 1.05 |  | 249 | 1.04 |  |
|  | CCG | 157 | 0.65 |  | 158 | 0.66 |  |
| Threonine(T) | ACU | 471 | 1.59 | 5.163849 | 467 | 1.58 | 5.142708 |
|  | ACC | 232 | 0.78 |  | 233 | 0.79 |  |
|  | ACA | 356 | 1.2 |  | 355 | 1.2 |  |
|  | ACG | 126 | 0.43 |  | 127 | 0.43 |  |
| Alanine(A) | GCU | 588 | 1.85 | 5.542967 | 590 | 1.85 | 5.547337 |
|  | GCC | 186 | 0.58 |  | 184 | 0.58 |  |
|  | GCA | 340 | 1.07 |  | 344 | 1.08 |  |
|  | GCG | 158 | 0.5 |  | 157 | 0.49 |  |
| Tyrosine(Y) | UAU | 703 | 1.64 | 3.730173 | 703 | 1.65 | 3.715628 |
|  | UAC | 153 | 0.36 |  | 151 | 0.35 |  |
| Histidine(H) | CAU | 390 | 1.45 | 2.340073 | 391 | 1.45 | 2.340759 |
|  | CAC | 147 | 0.55 |  | 147 | 0.55 |  |
| Glutamine(Q) | CAA | 636 | 1.56 | 3.551508 | 639 | 1.56 | 3.558998 |
|  | CAG | 179 | 0.44 |  | 179 | 0.44 |  |
| Asparagine(N) | AAU | 859 | 1.55 | 4.841381 | 861 | 1.54 | 4.859903 |
|  | AAC | 252 | 0.45 |  | 256 | 0.46 |  |
| Lysine(K) | AAA | 938 | 1.56 | 5.25536 | 940 | 1.55 | 5.264532 |
|  | AAG | 268 | 0.44 |  | 270 | 0.45 |  |
| Aspartic acid(D) | GAU | 724 | 1.62 | 3.887049 | 723 | 1.62 | 3.889662 |
|  | GAC | 168 | 0.38 |  | 171 | 0.38 |  |
| Glutamic acid (E) | GAA | 922 | 1.52 | 5.294579 | 921 | 1.52 | 5.286286 |
|  | GAG | 293 | 0.48 |  | 294 | 0.48 |  |
| Cysteine (C) | UGU | 199 | 1.59 | 1.093777 | 197 | 1.58 | 1.083362 |
|  | UGC | 52 | 0.41 |  | 52 | 0.42 |  |
| Tryptophan (W) | UGG | 396 | 1 | 1.725641 | 397 | 1 | 1.727289 |
| Arginine (R) | CGU | 310 | 1.36 | 5.948231 | 310 | 1.36 | 5.938914 |
|  | CGC | 91 | 0.4 |  | 90 | 0.4 |  |
|  | CGA | 309 | 1.36 |  | 310 | 1.36 |  |
|  | CGG | 107 | 0.47 |  | 108 | 0.47 |  |
|  | AGA | 391 | 1.72 |  | 389 | 1.71 |  |
|  | AGG | 157 | 0.69 |  | 158 | 0.69 |  |
| Glysine(G) | GGU | 537 | 1.35 | 6.915635 | 539 | 1.36 | 6.904803 |
|  | GGC | 181 | 0.46 |  | 179 | 0.45 |  |
|  | GGA | 570 | 1.44 |  | 570 | 1.44 |  |
|  | GGG | 299 | 0.75 |  | 299 | 0.75 |  |
| Stop codon(*) | UAA | 50 | 1.85 | 0.352972 | 49 | 1.81 | 0.352419 |
|  | UAG | 18 | 0.67 |  | 19 | 0.7 |  |
|  | UGA | 13 | 0.48 |  | 13 | 0.48 |  |

**Supplementary Table S3a.** List and location of long repeat sequences in the chloroplast genome of *Alchemilla* *argyrophylla*

| Repeat size (bp) | 1^st^ start | Repeat type | 2^nd^ start | location 1 | location 2 | Region |
| --- | --- | --- | --- | --- | --- | --- |
| 71 | 51202 | P | 51202 | IGS(*trnM-CAU-atpE*) | IGS(*trnM-CAU-atpE*) | LSC |
| 52 | 9435 | P | 9435 | IGS(*trnR-UCU-atpA*) | IGS(*trnR-UCU-atpA*) | LSC |
| 52 | 49977 | P | 49977 | IGS(*ndhC-trnV-UAC*) | IGS(*ndhC-trnV-UAC*) | LSC |
| 48 | 30565 | P | 30565 | IGS(*trnE-UUC-trnT-GGU*) | IGS(*trnE-UUC-trnT-GGU*) | LSC |
| 40 | 97043 | F | 118313 | IGS(*rps12-trnV-GAC*) | *ndhA* | IRb/SSC |
| 40 | 118313 | P | 137960 | *ndhA* | IGS(*trnV-GAC-rps12*) | SSC/IRa |
| 39 | 42988 | F | 97045 | *ycf3* | IGS(*rps12-trnV-GAC*) | LSC/IRb |
| 39 | 42988 | P | 137959 | *ycf3* | IGS(*trnV-GAC-rps12*) | LSC/IRa |
| 38 | 42988 | F | 118315 | *ycf3* | *ndhA* | LSC/SSC |
| 34 | 7844 | P | 44299 | IGS(*psbI-trnS-GCU*) | *trnS-GGA* | LSC |
| 34 | 55894 | F | 55925 | IGS(*rbcL-accD*) | IGS(*rbcL-accD*) | LSC |
| 30 | 123110 | P | 123110 | *ycf1* | *ycf1* | SSC |
| 35 | 12177 | R | 12177 | IGS(*atpF-atpH*) | IGS(*atpF-atpH*) | LSC |
| 32 | 9781 | F | 9808 | IGS(*trnR-UCU-atpA*) | IGS(*trnR-UCU-atpA*) | LSC |
| 34 | 50131 | R | 50131 | IGS(*ndhC-trnV-UAC*) | IGS(*ndhC-trnV-UAC*) | LSC |
| 32 | 111873 | P | 111873 | IGS(*rpl32-trnL-UAG*) | IGS(*rpl32-trnL-UAG*) | SSC |
| 34 | 4242 | P | 12172 | IGS(*trnK-UUU -rps16*) | IGS(*atpF-atpH*) | LSC |
| 31 | 21239 | F | 79946 | *rpoC1* | *rpl16* | LSC |
| 31 | 111032 | P | 111066 | IGS(*ndhF-rpl32*) | IGS(*ndhF-rpl32*) | SSC |
| 33 | 12179 | R | 12189 | IGS(*atpF-atpH*) | IGS(*atpF-atpH*) | LSC |
| 33 | 34695 | P | 44298 | IGS(*psbC-trnS-UGA*) | *trnS-GGA* | LSC |
| 30 | 12179 | R | 12179 | IGS(*atpF-atpH*) | IGS(*atpF-atpH*) | LSC |
| 30 | 12179 | F | 12180 | IGS(*atpF-atpH*) | IGS(*atpF-atpH*) | LSC |
| 30 | 12180 | R | 12180 | IGS(*atpF-atpH*) | IGS(*atpF-atpH*) | LSC |
| 30 | 63683 | R | 63683 | IGS(*psbE-petL*) | IGS(*psbE-petL*) | LSC |
| 30 | 105850 | F | 105882 | IGS(*rrn4.5-rrn5*) | IGS(*rrn4.5-rrn5*) | IRb |
| 30 | 105850 | P | 129131 | IGS(*rrn4.5-rrn5*) | IGS(*rrn5-rrn4.5*) | IRb /IRa |
| 30 | 105882 | P | 129163 | IGS(*rrn4.5-rrn5*) | IGS(*rrn5-rrn4.5*) | IRb /IRa |
| 30 | 129131 | F | 129163 | IGS(*rrn5-rrn4.5*) | IGS(*rrn5-rrn4.5*) | IRa |
| 32 | 7846 | F | 34695 | IGS(*psbl-trnS-GCU*), *trnS-GCU* | IGS(*psbC-trnS-UGA*), *trnS-UGA* | LSC |
| 32 | 12177 | R | 29165 | IGS(*atpF-atpH*) | IGS(*psbM-trnD-GUC*) | LSC |
| 32 | 12179 | F | 12181 | IGS(*atpF-atpH*) | IGS(*atpF-atpH*) | LSC |
| 31 | 12179 | R | 12193 | IGS(*atpF-atpH*) | IGS(*atpF-atpH*) | LSC |
| 31 | 94405 | P | 94405 | *ndhB* | *ndhB* | IRb |
| 31 | 94405 | F | 140607 | *ndhB* | *ndhB* | IRb/IRa |
| 31 | 140607 | P | 140607 | *ndhB* | *ndhB* | IRa |
| 30 | 4240 | P | 12179 | IGS(*trnK-UUU-rps16*) | IGS(*atpF-atpH*) | LSC |
| 30 | 4708 | P | 4848 | IGS(*trnK-UUU-rps16*) | IGS(*trnK-UUU-rps16*) | LSC |
| 30 | 9011 | F | 35711 | *trnG-UCC* | *trnG-GCC* | LSC |
| 30 | 9699 | F | 34931 | IGS(*trnR-UCU-atpA*) | IGS(*trnS-UGA-psbZ*) | LSC |
| 30 | 12179 | F | 12182 | IGS(*atpF-atpH*) | IGS(*atpF-atpH*) | LSC |

**Supplementary Table S3b.** List and location of long repeat sequences in the chloroplast genome of *Alchemilla pedata*

| Repeat size (bp) | 1^st^ start | Repeat type | 2^nd^ start | location 1 | location2 | region |
| --- | --- | --- | --- | --- | --- | --- |
| 71 | 51202 | P | 51202 | IGS(*trnM-CAU-atpE*) | IGS(*trnM-CAU-atpE*) | LSC |
| 52 | 9439 | P | 9439 | IGS(*trnR-UCU-atpA*) | IGS(*trnR-UCU-atpA*) | LSC |
| 48 | 30536 | P | 30536 | IGS(*trnE-UUC-trnT-GGU*) | IGS(*trnE-UUC-trnT-GGU*) | LSC |
| 40 | 49996 | P | 49996 | IGS(*ndhC-trnV-UAC*) | IGS(*ndhC-trnV-UAC*) | LSC |
| 40 | 97046 | F | 118328 | IGS(*rps12-trnV-GAC*) | *ndhA* | IRb/SSC |
| 40 | 118328 | P | 137964 | *ndhA* | IGS(*trnV-GAC-rps12*) | SSC/IRa |
| 39 | 42976 | F | 97048 | *ycf3* | IGS(*rps12-trnV-GAC*) | LSC/IRb |
| 39 | 42976 | P | 137963 | *ycf3* | IGS(*trnV-GAC-rps12*) | LSC/IRa |
| 38 | 42976 | F | 118330 | *ycf3* | *ndhA* | LSC/SSC |
| 34 | 7875 | P | 44317 | IGS(*psbI-trnS-GCU*) | *trnS-GGA* | LSC |
| 34 | 55894 | F | 55925 | IGS(*rbcL-accD*) | IGS(*rbcL-accD*) | LSC |
| 30 | 43402 | F | 43430 | IGS(*trnS-GGA-rps4*) | IGS(*trnS-GGA-rps4*) | LSC |
| 30 | 123113 | P | 123113 | *ycf1* | *ycf1* | SSC |
| 35 | 27849 | F | 27865 | IGS(*petN-psbM*) | IGS(*petN-psbM*) | LSC |
| 34 | 4254 | R | 4254 | IGS(*trnK-UUU-rps16*) | IGS(*trnK-UUU-rps16*) | LSC |
| 33 | 111028 | P | 111064 | *ndhF* | *ndhF* | SSC |
| 32 | 111872 | P | 111872 | IGS(*ndhF-rpl32*) | IGS(*ndhF-rpl32*) | SSC |
| 31 | 4255 | R | 4255 | IGS(*trnK-UUU -rps16*) | IGS(*trnK-UUU-rps16*) | LSC |
| 31 | 4256 | R | 4256 | IGS(*trnK-UUU -rps16*) | IGS(*trnK-UUU-rps16*) | LSC |
| 31 | 21245 | F | 79937 | *rpoC1* | *rpl16* | LSC |
| 30 | 63679 | R | 63679 | IGS(*psbE-petL*) | IGS(*psbE-petL*) | LSC |
| 30 | 105854 | F | 105886 | IGS(*rrn4.5-rrn5*) | IGS(*rrn4.5-rrn5*) | IRb |
| 30 | 105854 | P | 129134 | IGS(*rrn4.5-rrn5*) | IGS(*rrn5-rrn4.5*) | IRb/IRa |
| 30 | 105886 | P | 129166 | IGS(*rrn4.5-rrn5*) | IGS(*rrn5-rrn4.5*) | IRb/IRa |
| 30 | 129134 | F | 129166 | IGS(*rrn5-rrn4.5*) | IGS(*rrn5-rrn4.5*) | IRa |
| 32 | 7877 | F | 34664 | IGS(*psbI-trnsS-GCU*),*trnS-GCU* | IGS(*psbC-trnS-UGA*), *trnS-UGA* | LSC |
| 32 | 34664 | P | 44317 | IGS(*psbC-trnS-UGA*),*trnS-UGA* | *trnS-GGA* | LSC |
| 31 | 4255 | F | 4256 | IGS(*trnK-UUU-rps16*) | IGS(*trnK-UUU -rps16*) | LSC |
| 31 | 49986 | P | 50035 | IGS(*ndhC-trnV-UAC*) | IGS(*ndhC-trnV-UAC*) | LSC |
| 31 | 94408 | P | 94408 | *ndhB* | *ndhB* | IRb |
| 31 | 94408 | F | 140611 | *ndhB* | *ndhB* | IRb/IRa |
| 31 | 140611 | P | 140611 | *ndhB* | *ndhB* | IRa |
| 30 | 6529 | P | 35606 | IGS(*rps16-trnQ-UUG*) | IGS(*psbZ-trnG-GCC*) | LSC |
| 30 | 9044 | F | 35686 | *trnG-UCC* | *trnG-GCC* | LSC |
| 30 | 11531 | F | 78834 | IGS(*atpA-atpF*) | IGS(*rps8-rpl14*) | LSC |
|  |  |  |  |  |  |  |

**Supplementary Table S4a.** Tandem repeats units in *Alchemilla argyrophylla*

| Indices | Period size | Matches | Mismatch | Indels | Repeat unit | Location |
| --- | --- | --- | --- | --- | --- | --- |
| 240-285 | 23 | 21 | 2 | 0 | GTCTTATCCATTAATAAATGGAA | IGS(*trnH-GUG - psbA*), *psbA* |
| 6097-6129 | 16 | 17 | 0 | 0 | TAGTAATCTATACCTT | IGS(*rps16 - trnQ-UUG*) |
| 6777-6811 | 13 | 19 | 1 | 2 | ACTAATAAATAAAC | IGS(*trnQ-UUG - psbK)* |
| 9093-9135 | 20 | 23 | 0 | 0 | TTTTTTTAAATATTCAATAC | IGS(*trnG-UCC* - *trnR-UCU)* |
| 9772-9827 | 27 | 25 | 4 | 4 | TTATAAATTAATAAGTTTTTTTTAG | IGS(*trnR-UCU - atpA)* |
| 9790-9836 | 14 | 28 | 4 | 2 | TTTTTAGTTATTTA | IGS(*trnR-UCU* - *atpA*) |
| 9799-9840 | 13 | 24 | 4 | 2 | ATTTATTTTTATA | IGS(*trnR-UCU*-*atpA*) |
| 9782-9840 | 27 | 31 | 1 | 0 | ATAATTTTTTTTTAGTTATTTATTTTT | IGS(*trnR-UCU*-*atpA*) |
| 12138-12170 | 17 | 15 | 1 | 0 | CTTTCTTATAGTTATAG | IGS(*atpF - atpH)* |
| 12181-12227 | 20 | 24 | 2 | 1 | TTTTTTTTTTATAGTCTTTTT | IGS(*atpF* - *atpH*) |
| 12180-12210 | 1 | 28 | 2 | 0 | T | IGS(*atpF* - *atpH*) |
| 26137-26167 | 15 | 16 | 0 | 0 | AATTATTTAATAAAC | IGS(*rpoB* - *trnC-GCA*) |
| 27854-27888 | 16 | 18 | 0 | 0 | AAAGTAAAAAGAAATAC | IGS(*petN* - *psbM*) |
| 28333-28373 | 7 | 30 | 2 | 4 | AGAAATT | IGS(*petN* - *psbM*) |
| 28327-28373 | 23 | 24 | 0 | 0 | AAATACAGAAATTAGAAATTAGA | IGS(*petN* - *psbM*) |
| 34430-34459 | 15 | 15 | 0 | 0 | CTCCTCTTAACTGAG | *psbC,* IGS(*psbC* - *trnS-UGS*) |
| 45817-45846 | 15 | 15 | 0 | 0 | AGAAAGGAATAAAAA | IGS(*trnT* - *trnL-UAA*) |
| 50100-50135 | 14 | 22 | 0 | 4 | CTTTAATTTATT | IGS(*ndhC* - trnV-UAC) |
| 50101-50229 | 14 | 15 | 0 | 0 | TTTAATTTATTCTT | IGS(*ndhC* - *trnV-UAC*), *trnV-UAC* |
| 50094-50165 | 17 | 47 | 2 | 15 | ATTTAATTTAATTT | IGS(*ndhC* - *trnV-UAC*) |
| 50083-50165 | 26 | 47 | 6 | 9 | TAATTTATTTAATTTAACTTTAATT | IGS(*ndhC* - *trnV-UAC*) |
| 55273-55310 | 19 | 19 | 0 | 0 | AATTTGAAGCAATGGATAC | *rbcL,* IGS(*rbcL - accD*) |
| 55895-55959 | 31 | 33 | 1 | 0 | AATGACTATTCATCTATTGTATTTTCATGTA | IGS(*rbcL* - *accD*) |
| 57641-57675 | 11 | 22 | 2 | 0 | TTATTATAATA | IGS(*accD* - *psaI*) |
| 63362-63388 | 9 | 18 | 0 | 0 | CCAGTACTA | IGS(*psbE* - *petL*) |
| 63381-63418 | 17 | 20 | 1 | 1 | CAGTACTAATAGGAAAT | IGS(*psbE* - *petL*) |
| 72671-72706 | 18 | 18 | 0 | 0 | ATTATATAGTTTTAAGAC | IGS(psbN - *psbH*) |
| 87395-87462 | 21 | 37 | 10 | 0 | TTTTTGTCCAAGTTACTTCTT | ycf2 |
| 89829-89865 | 18 | 19 | 0 | 0 | TTGATGATAGTGACGATA | ycf2 |
| 105851-105912 | 32 | 28 | 2 | 0 | CATTGTTCAACTCTTTGACAACACGAAAAAAC | IGS(*rrn4.5 -rrn5)* |
| 106925-106952 | 14 | 14 | 0 | 0 | CATTCCGAATTAAA | IGS(*trnR-ACG* - *trnN-GUU)* |
| 111009-111048 | 17 | 22 | 1 | 1 | ATTAATATAAATTAAAT | IGS(*ndhF - rpl32)* |
| 111004-111102 | 32 | 58 | 0 | 19 | TTTAAATTAATTAAATTAATTTAATATAAA | IGS(*ndhF* - *rpl32*) |
| 111037-111091 | 28 | 25 | 0 | 4 | TTAATATTGAATTAAATTATTAATATTG | IGS(*ndhF* - *rpl32*) |
| 111013-111096 | 19 | 53 | 8 | 9 | ATTAAATTAAATATTAAT | IGS(*ndhF* - *rpl32*) |
| 128081-128118 | 14 | 20 | 2 | 2 | ATTCGGAATTCTTTA | IGS(*trnN-GUU - trnR-ACG)* |
| 128092-128119 | 14 | 14 | 0 | 0 | TTTAATTCGGAATG | IGS(*trnN-GUU* - *trnR-ACG*) |
| 129132-129193 | 32 | 28 | 2 | 0 | TTTTTCATGTTGTCAAAGAGCTGAACAATGGT | IGS(*rrn5* - *rrn 4.5*) |
| 145179-145215 | 18 | 19 | 0 | 0 | ATATCGTCACTATCATCA | *ycf2* |
| 147600-147651 | 21 | 27 | 4 | 0 | GACAAAAAGAGAAGAAACCTG | *ycf2* |

IGS: Intergenic spacers

**Supplementary Table S4b.** Tandem repeats units in *Alchemilla pedata*

| Indices | Period Size | Matches | Mismatch | Indels | Repeat Units | Location |
| --- | --- | --- | --- | --- | --- | --- |
| 248-293 | 23 | 21 | 2 | 0 | GTCTTATCCATTAATAAATGGAA | IGS(*trnH-GuG* - *psbA*), *psbA* |
| 6117-6149 | 16 | 17 | 0 | 0 | TAGTAATCTATACCTT | IGS(*rps16* - *trnQ-UUG*) |
| 6805-6839 | 13 | 19 | 1 | 2 | ACTAATAAATAAAC | IGS(*trnQ-UUG* -*psbK*) |
| 9624-9655 | 16 | 16 | 0 | 0 | TTTGATTATTTTAATT | IGS(*trnR-UCU*- *atpA*) |
| 9644-9686 | 19 | 20 | 0 | 4 | ATTATTTAATTAGAGTACTCT | IGS(*trnR-UCU*-*atpA*) |
| 9650-9690 | 19 | 22 | 0 | 0 | TTAATTAGAGTACTCTATA | IGS(*trnR-UCU*-*atpA*) |
| 12150-12182 | 17 | 15 | 1 | 0 | CTTTCTTATAGTTATAG | IGS(*atpF* - *atpH*) |
| 12175-12228 | 24 | 25 | 5 | 1 | TATTTATATATAGACTTTTTTTT | IGS(*atpF* - *atpH*) |
| 14181-14219 | 20 | 19 | 0 | 1 | ATTTTAAAATTATATATTTT | IGS(*atpI* - *rps2*) |
| 26144-26174 | 15 | 16 | 0 | 0 | AATTATTTAATAAAC | IGS(*rpoB* - *trnC-GCA*) |
| 27850-27900 | 16 | 34 | 0 | 2 | AAAGTAAAAGAAATAC | IGS(*petN* - *psbM*) |
| 34399-34428 | 15 | 15 | 0 | 0 | CTCCTCTTAACTGAG | *psbC*, IGS(*psbC* - *trnS-UGS*) |
| 35427-35460 | 16 | 16 | 2 | 0 | GAAAAAAAAAAAAGTA | IGS(*psbZ* - *trnG-GCC*) |
| 41507-41552 | 9 | 33 | 0 | 7 | TTATTTTAG | IGS(*psbA - ycf3)* |
| 41512-41553 | 21 | 21 | 0 | 0 | TTTAGTTATTTTAGTTATTTG | IGS(*psbA* - *ycf3*) |
| 43403-43460 | 28 | 30 | 0 | 0 | TATTGGTACTAGTGAAGTAGGGTTAACT | *ycf3* (intron) |
| 45838-45867 | 15 | 15 | 0 | 0 | AGAAAGGAATAAAAA | IGS(*trnT-UGU* - *trnL-UAA*) |
| 50016-50056 | 20 | 21 | 0 | 0 | ATATAGATATATTAGAAATA | IGS(*ndhC* - *trnV-UAC*) |
| 50127-50169 | 21 | 19 | 2 | 1 | ATTTAACTTTAATATTAAATTT | IGS(*ndhC* - *trnV-UAC*) |
| 55273-55310 | 19 | 19 | 0 | 0 | AATTTGAAGCAATGGATAC | *rbcL*, IGS(*rbcL* - *accD*) |
| 55895-55959 | 31 | 33 | 1 | 0 | AATGACTATTCATCTATTGTATTTTCATGTA | IGS(*rbcL* - *accD*) |
| 57648-57682 | 11 | 22 | 2 | 0 | TTATTATAATA | IGS(*accD* - *psaI*) |
| 63379-63416 | 17 | 20 | 1 | 1 | CAGTACTAATAGGAAAT | IGS(*psbE* - *petL*) |
| 72649-72684 | 18 | 18 | 0 | 0 | ATTATATAGTTTTAAGAC | IGS(*psbN* - *psbH*) |
| 75909-75954 | 23 | 23 | 0 | 0 | CCCTAACTTTAGGTCTTTTTTAA | *petD*, IGS(*petD* - *rpoA*) |
| 87392-87459 | 21 | 37 | 10 | 0 | TTTTTGTCCAAGTTACTTCTT | *ycf2* |
| 89826-89862 | 18 | 19 | 0 | 0 | TTGATGATAGTGACGATA | *ycf2* |
| 105855-105916 | 32 | 28 | 2 | 0 | CATTGTTCAACTCTTTGACAACACGAAAAAAC | IGS(*rrn4.5* - *rrn5*) |
| 106929-106956 | 14 | 14 | 0 | 0 | CATTCCGAATTAAA | IGS(*trnR-ACG* - *trnN-GUU*) |
| 111011-111045 | 17 | 17 | 1 | 0 | TTTAAATTCAATATTAA | IGS(*ndhF* - *rpl32*) |
| 111023-111089 | 28 | 37 | 0 | 7 | ATTAATTTAATATTGTTAATATTAATAA | IGS(*ndhF* - *rpl32*) |
| 111012-111104 | 19 | 61 | 10 | 8 | TTAAATTAAATTATTAATA | IGS(*ndhF* - *rpl32*) |
| 128084-128121 | 14 | 20 | 2 | 2 | ATTCGGAATTCTTTA | IGS(*trnN-GUU* - *trnR-ACG*) |
| 128095-128122 | 14 | 14 | 0 | 0 | TTTAATTCGGAATG | IGS(*trnN-GUU* - *trnR-ACG*) |
| 129135-129196 | 32 | 28 | 2 | 0 | TTTTTCATGTTGTCAAAGAGCTGAACAATGGT | IGS*(rrn5* - *rrn 4.5*) |
| 145189-145225 | 18 | 19 | 0 | 0 | ATATCGTCACTATCATCA | *ycf2* |
| 147610-147661 | 21 | 27 | 4 | 0 | GACAAAAAGAGAAGAAACCTG | *ycf2* |

IGS: Intergenic spacers

**Supplementary table S5**: synonymous and non-synonymous, and Ka/Ks ratio in *Alchemilla argyrophylla* and *Alchemilla* *pedata* with *Fragaria virginiana* as reference genome.

|  |  |  |  | Alchemilla Argyrophylla |  |  | Alchemilla pedata |  |  |
| --- | --- | --- | --- | --- | --- | --- | --- | --- | --- |
| Family Name | Sequence | Gene length | Method | Ka | Ks | Ka/Ks | Ka | Ks | Ka/Ks |
| Small subunit of ribosome | rps2 | 708 | MA | 0.0000 | 0.0442 | 0.0010 | 0.0000 | 0.0442 | 0.0010 |
|  | rps3 | 654 | MA | 0.0061 | 0.0737 | 0.0824 | 0.0060 | 0.0657 | 0.0912 |
|  | rps4 | 603 | MA | 0.0024 | 0.0961 | 0.0251 | 0.0001 | 0.1089 | 0.0010 |
|  | rps7 | 465 | MA | 0.0027 | 0.0001 | 0.0000 | 0.0028 | 0.0105 | 0.2645 |
|  | rps8 | 402 | MA | 0.0099 | 0.0921 | 0.1074 | 0.0101 | 0.1185 | 0.0849 |
|  | rps11 | 414 | MA | 0.0031 | 0.0795 | 0.0396 | 0.0031 | 0.0795 | 0.0396 |
|  | rps12 | 369 | MA | 0.0036 | 0.0124 | 0.2880 | 0.0036 | 0.0124 | 0.2880 |
|  | rps14 | 300 | MA | 0.0042 | 0.0494 | 0.0848 | 0.0042 | 0.0494 | 0.0848 |
|  | rps15 | 270 | MA | 0.0111 | 0.0779 | 0.1428 | 0.0111 | 0.0779 | 0.1428 |
|  | rps16 | 267 | MA | 0.0089 | 0.1094 | 0.0815 | 0.0089 | 0.1094 | 0.0815 |
|  | rps18 | 303 | MA | 0.0132 | 0.0299 | 0.4411 | 0.0132 | 0.0299 | 0.4411 |
|  | rps19 | 276 | MA | 0.0001 | 0.0995 | 0.0010 | 0.0001 | 0.0839 | 0.0010 |
| Large subunit of ribosome | rpl2 | 822 | MA | 0.0015 | 0.0115 | 0.1315 | 0.0015 | 0.0115 | 0.1315 |
|  | rpl14 | 366 | MA | 0.0036 | 0.1481 | 0.0245 | 0.0036 | 0.1481 | 0.0245 |
|  | rpl16 | 405 | MA | 0.0061 | 0.0752 | 0.0809 | 0.0061 | 0.0752 | 0.0809 |
|  | rpl20 | 351 | MA | 0.0042 | 0.0813 | 0.0517 | 0.0042 | 0.0813 | 0.0517 |
|  | rpl22 | 417 | MA | 0.0186 | 0.0625 | 0.2970 | 0.0212 | 0.0648 | 0.3270 |
|  | rpl23 | 279 | MA | 0.0044 | 0.0001 | 0.0000 | 0.0044 | 0.0001 | 0.0000 |
|  | rpl32 | 156 | MA | 0.0096 | 0.0512 | 0.1875 | 0.0096 | 0.0512 | 0.1875 |
|  | rpl33 | 198 | MA | 0.0201 | 0.0626 | 0.3206 | 0.0136 | 0.0612 | 0.2215 |
|  | rpl36 | 111 | MA | 0.0002 | 0.1900 | 0.0010 | 0.0002 | 0.1900 | 0.0010 |
| DNA dependent RNA polymerase | rpoA | 993 | MA | 0.0140 | 0.1054 | 0.1325 | 0.0140 | 0.1054 | 0.1325 |
|  | rpoB | 3210 | MA | 0.0046 | 0.0694 | 0.0660 | 0.0042 | 0.0690 | 0.0607 |
|  | rpoC1 | 2040 | MA | 0.0037 | 0.0809 | 0.0461 | 0.0031 | 0.0841 | 0.0368 |
|  | rpoC2 | 4149 | MA | 0.0173 | 0.0868 | 0.1989 | 0.0173 | 0.0865 | 0.1995 |
| Subunit of ATP synthase | atpA | 1521 | MA | 0.0030 | 0.0753 | 0.0393 | 0.0030 | 0.0721 | 0.0410 |
|  | atpB | 1476 | MA | 0.0025 | 0.0805 | 0.0312 | 0.0025 | 0.0816 | 0.0302 |
|  | atpE | 399 | MA | 0.0033 | 0.0886 | 0.0375 | 0.0033 | 0.0886 | 0.0374 |
|  | atpF | 552 | MA | 0.0067 | 0.1156 | 0.0582 | 0.0067 | 0.1173 | 0.0571 |
|  | atpH | 243 | MA | 0.0001 | 0.0599 | 0.0010 | 0.0001 | 0.0554 | 0.0010 |
|  | atpI | 741 | MA | 0.0033 | 0.0875 | 0.0374 | 0.0050 | 0.0923 | 0.0541 |
| Subunit of NADH-dehydrogenase | ndhA | 1080 | MA | 0.0121 | 0.0592 | 0.2051 | 0.0111 | 0.0629 | 0.1759 |
|  | ndhB | 1530 | MA | 0.0018 | 0.0175 | 0.1054 | 0.0018 | 0.0175 | 0.1054 |
|  | ndhC | 360 | MA | 0.0083 | 0.0605 | 0.1371 | 0.0083 | 0.0735 | 0.1136 |
|  | ndhD | 1500 | MA | 0.0155 | 0.0729 | 0.2125 | 0.0155 | 0.0748 | 0.2071 |
|  | ndhE | 300 | MA | 0.0084 | 0.1306 | 0.0647 | 0.0084 | 0.1703 | 0.0491 |
|  | ndhF | 2229 | MA | 0.0190 | 0.1533 | 0.1240 | 0.0191 | 0.1642 | 0.1164 |
|  | ndhG | 528 | MA | 0.0094 | 0.1200 | 0.0782 | 0.0118 | 0.1354 | 0.0870 |
|  | ndhH | 1179 | MA | 0.0011 | 0.1046 | 0.0105 | 0.0011 | 0.1069 | 0.0105 |
|  | ndhI | 501 | MA | 0.0001 | 0.1166 | 0.0010 | 0.0001 | 0.1166 | 0.0010 |
|  | ndhJ | 474 | MA | 0.0080 | 0.0811 | 0.0983 | 0.0081 | 0.0925 | 0.0879 |
|  | ndhK | 678 | MA | 0.0018 | 0.0607 | 0.0298 | 0.0018 | 0.0607 | 0.0298 |
| Subunit of cytochrome b/f complex | petA | 960 | MA | 0.0100 | 0.0690 | 0.1449 | 0.0114 | 0.0694 | 0.1648 |
|  | petB | 645 | MA | 0.0081 | 0.0382 | 0.2117 | 0.0081 | 0.0382 | 0.2117 |
|  | petD | 480 | MA | 0.0000 | 0.0463 | 0.0010 | 0.0001 | 0.0626 | 0.0010 |
|  | petG | 111 | MA | 0.0001 | 0.0791 | 0.0010 | 0.0001 | 0.0791 | 0.0010 |
|  | petL | 93 | MA | 0.0003 | 0.2642 | 0.0010 | 0.0003 | 0.2642 | 0.0010 |
|  | petN | 87 | MA | 0.0000 | 0.0000 | 0.7124 | 0.0000 | 0.0000 | 0.7124 |
| Subunit of photosystem I | psaA | 2250 | MA | 0.0030 | 0.0606 | 0.0491 | 0.0028 | 0.0612 | 0.0461 |
|  | psaB | 2202 | MA | 0.0017 | 0.0959 | 0.0175 | 0.0017 | 0.0929 | 0.0183 |
|  | psaC | 243 | MA | 0.0001 | 0.0860 | 0.0010 | 0.0001 | 0.0860 | 0.0010 |
|  | psaI | 111 | MA | 0.0131 | 0.0369 | 0.3546 | 0.0131 | 0.0369 | 0.3546 |
|  | psaJ | 126 | MA | 0.0126 | 0.0736 | 0.1712 | 0.0126 | 0.0736 | 0.1712 |
| Subunit of photosystem II | psbA | 1059 | MA | 0.0001 | 0.0640 | 0.0010 | 0.0001 | 0.0640 | 0.0010 |
|  | psbB | 1524 | MA | 0.0034 | 0.0739 | 0.0464 | 0.0034 | 0.0811 | 0.0419 |
|  | psbC | 1419 | MA | 0.0024 | 0.0573 | 0.0418 | 0.0031 | 0.0574 | 0.0547 |
|  | psbD | 1056 | MA | 0.0027 | 0.0707 | 0.0385 | 0.9414 | 1.2010 | 0.7839 |
|  | psbE | 249 | MA | 0.0068 | 0.0280 | 0.2410 | 0.0061 | 0.0521 | 0.1172 |
|  | psbF | 117 | MA | 0.0125 | 0.0335 | 0.3735 | 0.0125 | 0.0335 | 0.3735 |
|  | psbH | 219 | MA | 0.0001 | 0.1259 | 0.0010 | 0.0001 | 0.1259 | 0.0010 |
|  | psbI | 108 | MA | 0.0001 | 0.0623 | 0.0010 | 0.0001 | 0.0623 | 0.0010 |
|  | psbJ | 120 | MA | 0.0220 | 0.0005 | 0.0000 | 0.0220 | 0.0005 | 0.0000 |
|  | psbK | 183 | MA | 0.0093 | 0.0672 | 0.1380 | 0.0093 | 0.0672 | 0.1380 |
|  | psbL | 114 | MA | 0.0000 | 0.0000 | 0.7135 | 0.0000 | 0.0000 | 0.7135 |
|  | psbM | 102 | MA | 0.0000 | 0.0374 | 0.0010 | 0.0000 | 0.0374 | 0.0010 |
|  | psbN | 129 | MA | 0.0000 | 0.0000 | 0.6926 | 0.0000 | 0.0000 | 0.6926 |
|  | psbT | 99 | MA | 0.0000 | 0.0404 | 0.0010 | 0.0000 | 0.0404 | 0.0010 |
|  | psbZ | 186 | MA | 0.0075 | 0.0431 | 0.1739 | 0.0075 | 0.0431 | 0.1739 |
|  | rbcL | 1425 | MA | 0.0090 | 0.0843 | 0.1070 | 0.0098 | 0.0870 | 0.1130 |
| Protease | clpP | 585 | MA | 0.0024 | 0.0941 | 0.0251 | 0.0024 | 0.0941 | 0.0251 |
| Subunit of Acetyle-CoA-carboxylase | accD | 1476 | MA | 0.0249 | 0.1150 | 0.2162 | 0.0279 | 0.1016 | 0.2742 |
| Envelop membrane protein | cemA | 687 | MA | 0.0176 | 0.0856 | 0.2059 | 0.0172 | 0.0990 | 0.1737 |
| C-type cytochrome synthesis gene | ccsA | 957 | MA | 0.0188 | 0.1460 | 0.1287 | 0.0191 | 0.1366 | 0.1401 |
| Maturase | matK | 1500 | MA | 0.0289 | 0.1209 | 0.2389 | 0.0292 | 0.1144 | 0.2555 |
| Conserved open reading frames | ycf1 | 5694 | MA | 0.0411 | 0.1148 | 0.3577 | 0.0416 | 0.1177 | 0.3534 |
|  | ycf2 | 6825 | MA | 0.0055 | 0.0114 | 0.4867 | 0.0051 | 0.0124 | 0.4133 |
|  | ycf3 | 504 | MA | 0.0027 | 0.0358 | 0.0751 | 0.0027 | 0.0358 | 0.0751 |
|  | ycf4 | 552 | MA | 0.0113 | 0.0484 | 0.2332 | 0.0128 | 0.0462 | 0.2761 |

**Supplementary Table** **S6**: Comparative number of gene containing introns among Rosaceae species

| Taxa | *Potentilla chinensis* | *Rubus takesimensis* | *Fragaria Vesca subsp. Vesca* | *Pyrus spinosa* | *Prunus mume* | *Eriobotrya japonica* | *Alchemilla Pedata* | *Alchemilla argyrophylla* |
| --- | --- | --- | --- | --- | --- | --- | --- | --- |
| Total cpDNA size (bp) | 157,117 | 155,760 | 155,691 | 159,161 | 157,916 bp | 159,156 | 152,438 | 152427 |
| Number of single introns genes | 15 (lost *atpF* intron) | 15 (lost *atpF* intron) | 15 (lost *atpF* intron) | 16 | 16 | 16 | 15 (lost *atpF* intron) | 15 (lost *atpF* intron) |
| Number of two intron genes | 2 | 2 | 2 | 2 | 2 | 2 | 2 | 2 |
| Accession number | MN871983 | NC039379 | JF345175 | NC_023130 | MH00953 | MN577877 | MT382662 | MT382661 |


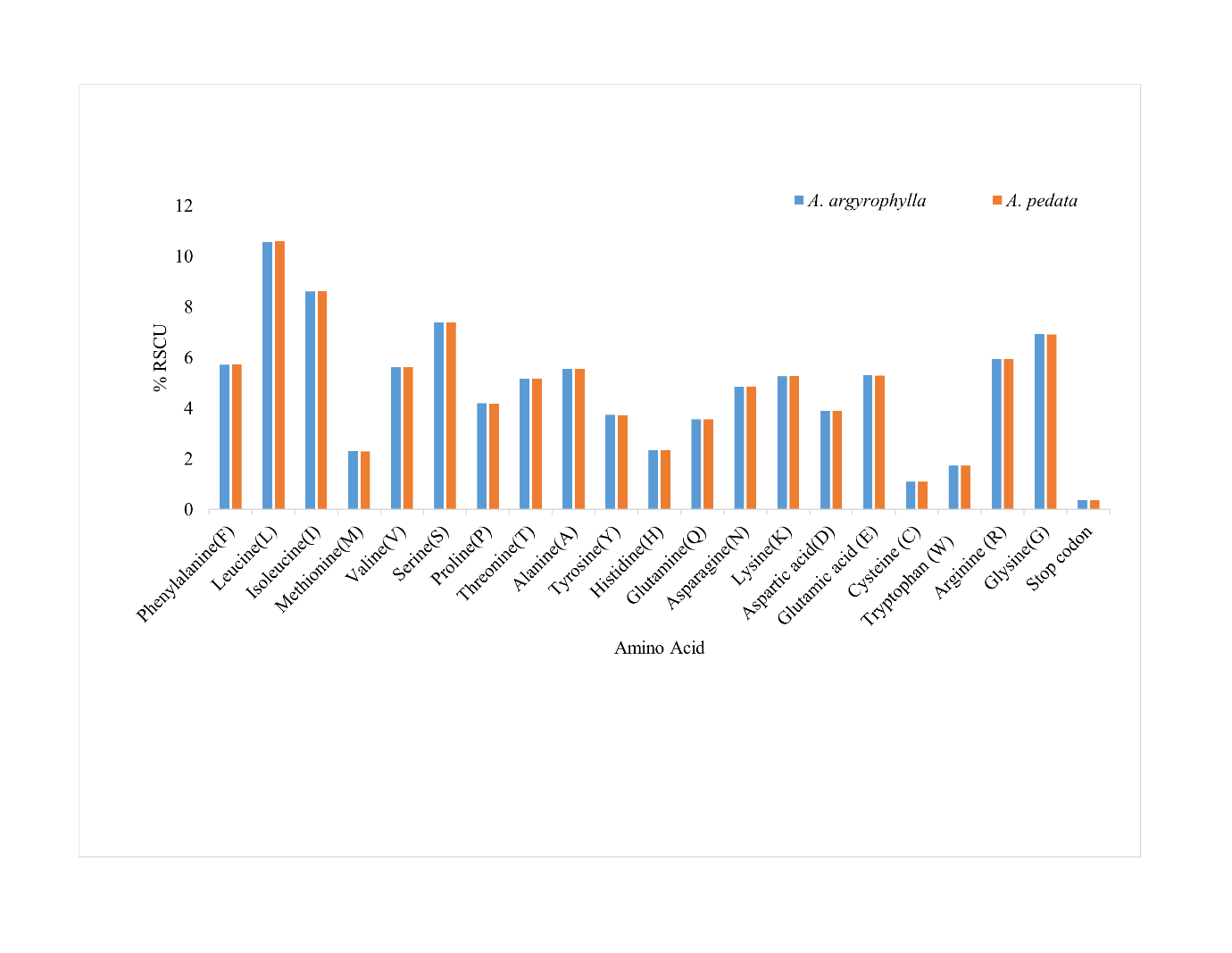


**Supplementary Figure S1**. Amino acid frequency usage in 88 protein-coding genes of *Alchemilla pedata* and *A. argyrophylla* chloroplast genomes. The Percentage Relative Synonymous Codon Usage (% RSCU) was calculated, excluding the introns, from the start codon to the stop codon.


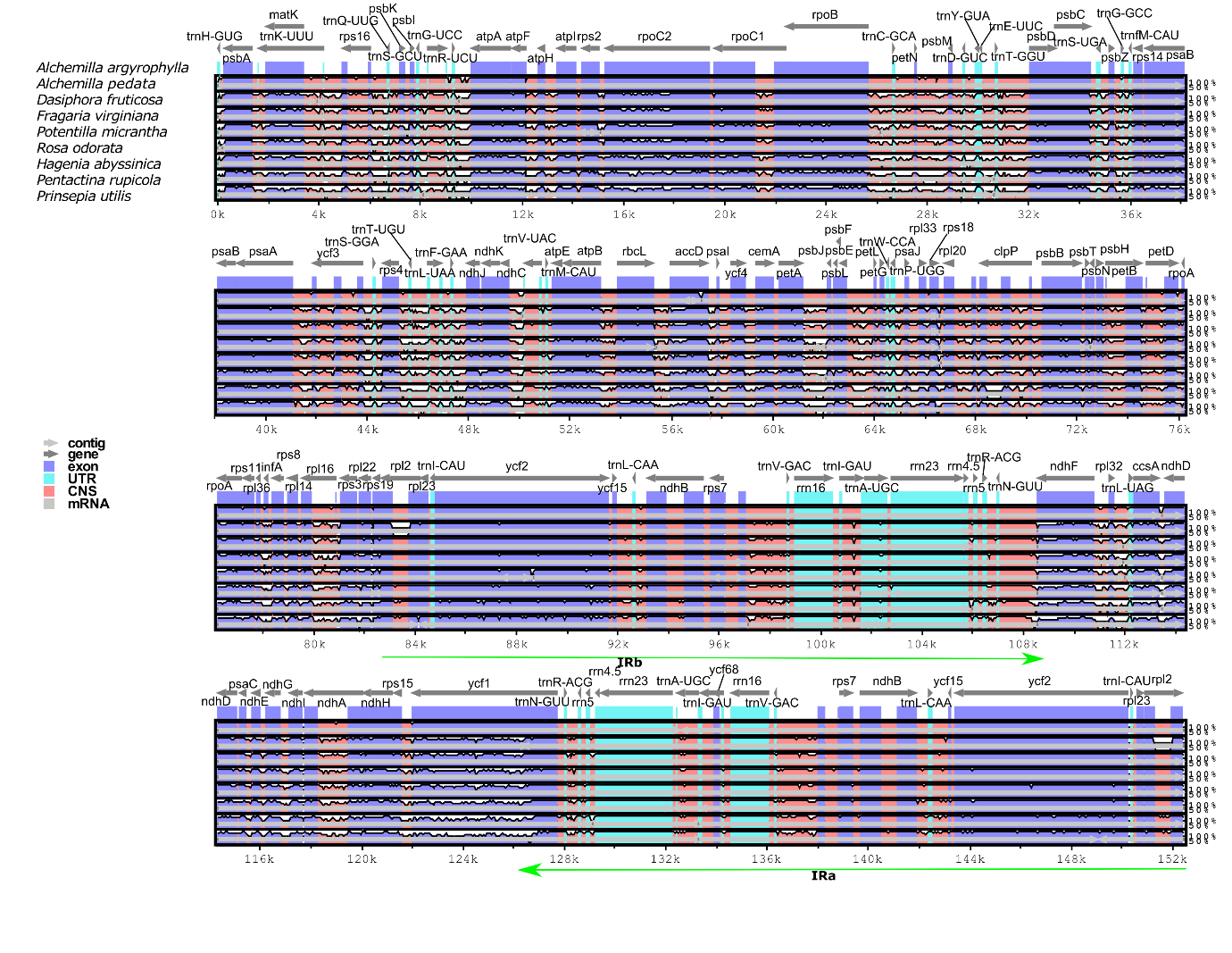


**Supplementary Figure S2**. Comparison of chloroplast genome of sequence identity in *Alchemilla argyrophylla, A. pedata* and seven other species within Rosaceae. The vertical axis indicates percentage identity ranging between 50% to 100% while position within the chloroplast genome is shown on the horizontal axis. The color codes denote mRNA, untranslated regions (UTR) and conserved noncoding sequences (CNS). The gray arrows display annotated genes and the direction of their transcription. Green arrows indicate the direction and position of the Inverted Repeats (IRa/IRb).
